# Photoredox Catalysts Based on *N*‐(Hexyl)benzothioxanthene‐3,4‐dicarboximide for Photopolymerization and 3D Printing Under Visible Light

**DOI:** 10.1002/anie.202501442

**Published:** 2025-04-25

**Authors:** Bin Song, Yijun Zhang, Zheng Liu, Pierre Boulay, Céline Dietlin, Fabrice Morlet‐Savary, Michael Schmitt, Didier Gigmes, Jean‐Michel Becht, Frédéric Dumur, Jacques Lalevée

**Affiliations:** ^1^ Université de Haute Alsace, CNRS, IS2M UMR 7361 Mulhouse F‐68100 France; ^2^ Université de Strasbourg Strasbourg France; ^3^ Aix Marseille Univ, CNRS, ICR UMR 7273 Marseilles F‐13397 France

**Keywords:** 3D Printing • Photocatalyst • Photochemistry • Photopolymerization • Photoredox catalysis

## Abstract

Photoredox catalytic systems are widely used in free radical polymerization as an important photoinitiating approach. However, many reported photoredox catalytic systems are limited by their low stabilities, high excitation powers, and low initiating efficiencies upon excitation in the visible region. Therefore, it is still a great challenge to develop efficient photoinitiating systems for photopolymerization under visible light. In this work, three new effective photosensitizers from *N*‐(hexyl)benzothioxanthene‐3,4‐dicarboximide derivatives, namely 2‐hexyl‐1*H*‐thioxantheno[2,1,9‐*def*]isoquinoline‐1,3(2*H*)‐dione (BTXI), 5‐bromo‐2‐hexyl‐1*H*‐thioxantheno[2,1,9‐*def*]isoquinoline‐1,3(2*H*)‐dione (BTXI‐Br) and 2‐hexyl‐1*H*‐thioxantheno[2,1,9‐*def*]isoquinoline‐1,3(2*H*)‐dione 6,6‐dioxide (BTXIO), were designed by density functional theory calculation and synthesized as photoredox catalysts for visible light induced photopolymerization. When combined with initiators such as oxidants, that is, *bis*(4‐*tert*‐butylphenyl)iodonium hexafluorophosphate or sulfonium salts (i.e., thianthrenium salts, phenoxathiinium salt, phenothiazinium salt, dibenzothiophenium salt) and the reductant ethyl dimethylaminobenzoate to form three‐component initiating systems, they showed good to high performance in visible light photo polymerizations with LED@405 nm and LED@450 nm. In addition, these photoinitiating systems enable the successful digital light processing and direct laser writing of 3D structures with high resolution, demonstrating a promising strategy for 3D printing applications.

## Graphical abstract

In this work, two benzothioxanthene imide dyes demonstrate excellent photochemical properties. When used as photocatalysts, they work with amine and iodonium salt or sulfonium salt to form different three‐component systems for effectively initiating photopolymerization under visible light, through photoredox catalysis processes. The best formulations can be applied for 3D printing and direct laser write. This work offers a strategy for developing novel photosensitive dyes as photocatalysts.

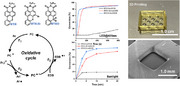

## Introduction

In recent decades, photopolymerization has garnered a significant research attention due to the continuous expansion of industrial applications driven by the development of new photoinitiators and monomers.^[^ ^1^, ^2^, ^3^, ^4^, ^5^ ^]^ Therefore, its applications can be evidently found not only in traditional fields such as coatings, inks and adhesives, but also in emerging research areas including stereolithography, biomaterials, 3D/4D printings, and nanotechnology.^[^ ^6^, ^7^, ^8^, ^9^, ^10^ ^]^ Besides, holographic photopolymerization for anticounterfeiting and augmented reality is an important field of photopolymerization as well.^[^ ^11^, ^12^, ^13^, ^14^ ^]^ In routine researches and production processes of academic and industrial fields, photoinitiating systems requiring high‐intensity UV light irradiation with substantial energy are still widely used. However, these photoinitiating systems are still facing safety and energy concerns.^[^ ^15^, ^16^ ^]^ To address these issues, an increasing number of photoinitiating systems sensitive to near‐UV and visible light regions have been developed and reported in recent years.^[^ ^17^, ^18^, ^19^ ^]^

Among those previous development in new photoinitiating systems, photoredox catalytic approaches have recently been rapidly developed in which a photocatalyst (PC) serves as a photosensitizer to promote the excitation of photoinitiators not sensitive to visible light.^[^ ^20^, ^21^, ^22^ ^]^ In our previous works,^[^ ^23^, ^24^, ^25^, ^26^, ^27^ ^]^ we reported various types of organic compounds as PCs, which worked with an iodonium salt [e.g., *bis*(4‐*tert*‐butylphenyl)iodonium hexafluorophosphate, (Iod)] and an organic base [e.g., an amine, i.e., ethyl dimethylaminobenzoate, (EDB)] to form a three‐component system that leads to the occurrence of a concomitant oxidative and reductive photocatalytic cycles to trigger the generation of free radicals, normally under LED@405 nm irradiation. In such systems, PCs usually work as photosensitizer to gain the initiating energy and then radicals and/or cationic species can be generated through photoredox catalytic process, which can initiate radical and/or cationic polymerization.^[^ ^28^, ^29^ ^]^ PCs can generally be classified into metal‐based and metal‐free catalysts.^[^ ^30^, ^31^ ^]^ However, metal‐based catalysts can be expensive especially the noble metals which are rare in nature, challenging to prepare, and in some cases with toxicity, which limit their applications in many fields especially in food packaging and medical materials, etc. In parallel, these limitations also drive the development of metal‐free catalysts, such as organic dyes sensitive to visible light from 380 nm up to 850 nm and even beyond, which show their green, economic and available characteristics.^[^ ^32^, ^33^, ^34^, ^35^, ^36^ ^]^

*N*‐(Hexyl)benzothioxanthene‐3,4‐dicarboximide, commonly referred to as benzothioxanthene imide (BTXI) for short,^[^ ^37^, ^38^ ^]^ a sulfur‐containing rylene‐imide dye, is widely recognized for its excellent fluorophore properties and this motif is extensively used for applications such as bioimaging, organic electronic devices or photosensitive materials.^[^ ^39^, ^40^, ^41^, ^42^ ^]^ BTXI typically exhibits a strong light absorption in visible light region between 400 and 500 nm.^[^ ^43^ ^]^ In our previous work,^[^ ^44^ ^]^ we introduced various oxime ester functional groups on the BTXI scaffold to synthesize BTXI‐based oxime ester compounds (BTXIOXEs). These compounds could be employed as Type I photoinitiators for free radical photopolymerizations through a photoinduced homolytic cleavage process. Additionally, BTXIOXEs also demonstrated a high efficiency when combined with Iod to form two‐component systems, generating radicals via a single electron transfer process, which also can be seen as an oxidation reaction. When the steady‐state photolysis experiments were performed on BTXIOXEs, they all exhibited a low consumption of 0%–5% after irradiation for 1500 s under LED@405 nm. Especially for BTXIOXE‐0, a BTXI‐based oxime, the consumption was close to 0% after 1500 s, which demonstrated its photochemical stability after a long‐time light irradiation. These findings highlight the potential of BTXI and its derivatives developed as PCs for photopolymerization, due to the excellent photochemical stability and its wide light absorption property.

**Scheme 1:**
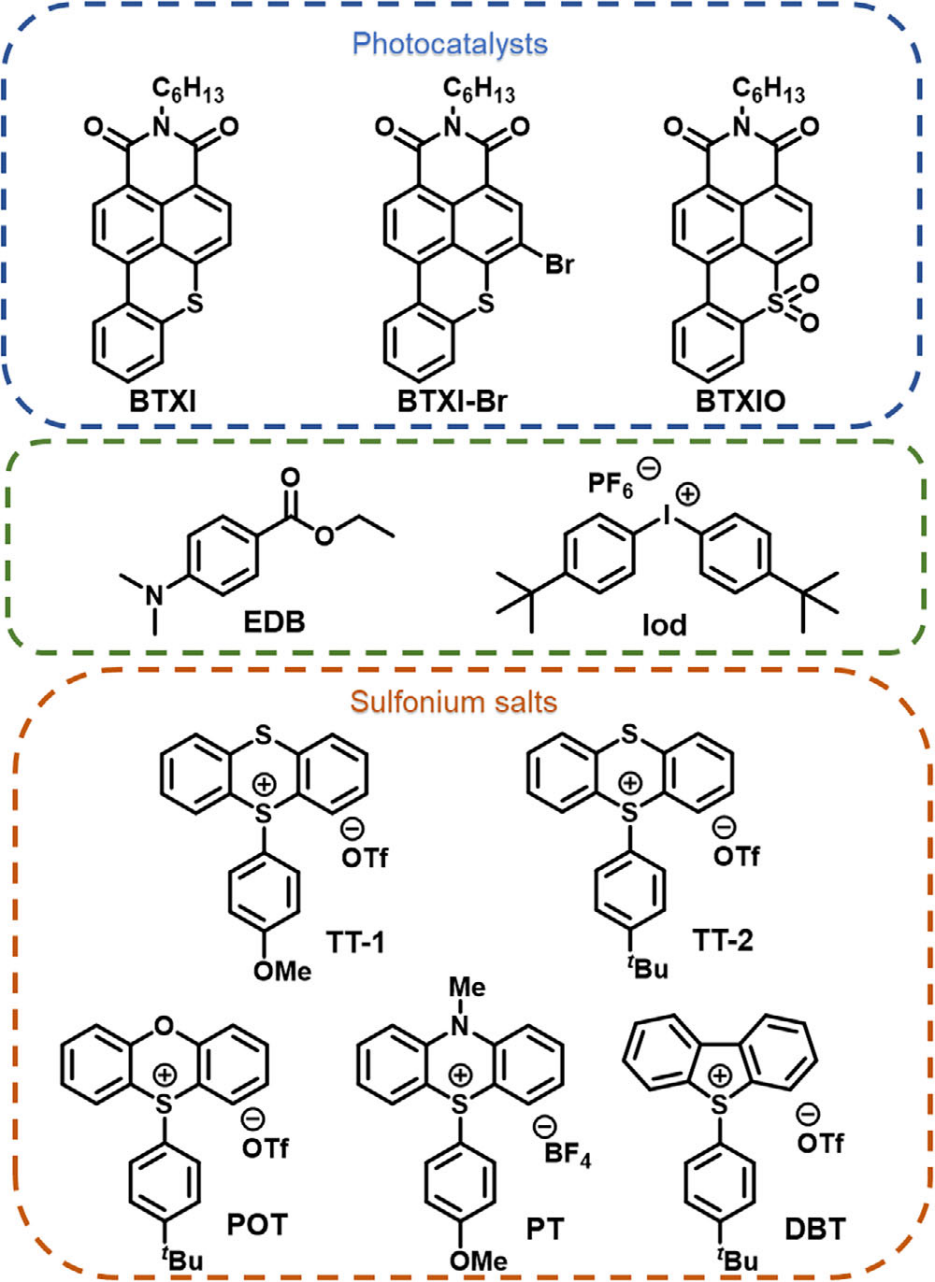
Chemical structures of PCs, EDB, Iod, and sulfonium salts (Sulfs).

Thus, in this work, three organic dyes, BTXI, a bromo derivative of BTXI (BTXI‐Br) and a BTXI‐based sulfone (BTXIO), were designed and synthesized to be PCs which presented excellent visible light absorption properties (see Scheme 1). In order to establish the photoredox catalytic systems, PCs were combined with Iod, five sulfonium salts (Sulfs), and EDB, to form two different three‐component catalytic systems: PC/Iod/EDB and PC/Sulf/EDB, respectively (See Scheme 1). The photochemical properties of three PCs, and the photoredox catalytic mechanisms of PC/Iod/EDB and PC/Sulf/EDB, were investigated in detail using density functional theory (DFT) calculation, steady‐state photolysis, fluorescence quenching and electron spin resonance (ESR) analyses. These photoinitiating systems exhibit a high photoinitiating efficiency in free radical photopolymerization. Furthermore, they were successfully applied in 3D printing and direct laser writing, producing high‐resolution objects.

## Results and Discussion

### Light Absorption Properties

**Figure 1:**
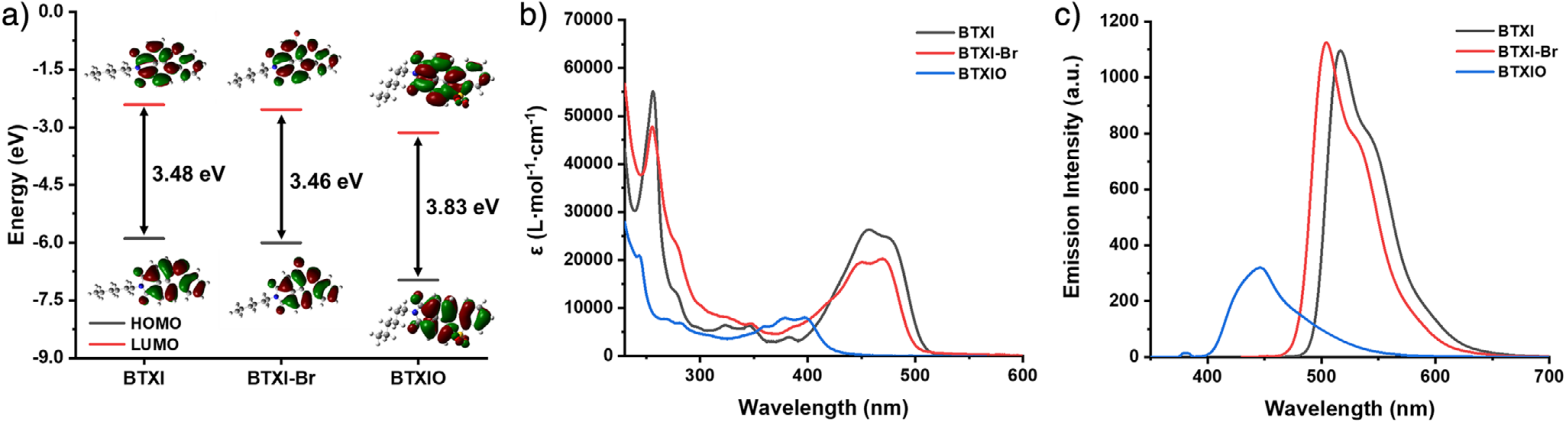
a) DFT calculation, b) UV‐visible absorption spectra, and (c) fluorescence emission spectra of BTXI, BTXI‐Br and BTXIO. The concentration of dyes in these experiments was controlled at 2 × 10^−5^ M in DCM.

DFT calculation was firstly performed on these dyes, to investigate the photocatalytic performance of dyes from a theoretical viewpoint. As shown in Figure 1a, the contour plots and the energy levels of the highest occupied molecular orbital (HOMO) and the lowest unoccupied molecular orbital (LUMO) of PCs were calculated. The HOMO and LUMO frontier orbitals of these PCs are similar, both mainly being distributed on the benzothioxanthene core, which demonstrate that these molecules could possess a high excited‐state stability. Compared to the bandgap of BTXI (△E_g _= 3.48 eV), BTXI‐Br exhibits a similar bandgap value of 3.46 eV, describing that the introduction of Br does not significantly affect its conjugation. Interestingly, BTXIO exhibits a larger bandgap value (△E_g _= 3.83 eV), suggesting its conjugation is weakened, and its light absorption properties could change accordingly, such as a potential blueshift in light absorption spectrum and a decrease in the molar extinction coefficient.

After obtaining these DFT calculation results, optical properties of dyes, Iod, and EDB were examined by UV‐visible absorption spectroscopy, respectively (see Figure 1b), and their photophysical characteristics are listed in Table 1. Both BTXI and BTXI‐Br show a high molar extinction coefficient (ε) from 400 to 500 nm and their maximum absorption wavelength (λ_max_) are 457 and 470 nm, respectively. The corresponding ε_max_ values of BTXI and BTXI‐Br are 26 200  and 21 000 M^−1^.cm^−1^, which indicate the possibility of photocatalysis in visible light mediated photopolymerization. However, the UV‐visible spectrum of BTXIO presents an obvious blueshift and its molar extinction coefficient is lower than both of BTXI and BTXI‐Br, that is, the λ_max_ of 397 nm and the ε_max_ of 7900 M^−1^ cm^−1^. This is consistent with the DFT calculation results of BTXIO. Typically, a LED@405 nm is a commonly used light source in photopolymerization. ε_405_ _nm_ of BTXI, BTXI‐Br, and BTXIO are 5600, 9100 and 6300 M^−1^.cm^−1^, respectively. Interestingly, BTXI may exhibit poor photoinitiating performance at a low concentration during photopolymerization due to the low molar extinction coefficient. Generally, Iod is used as an electron acceptor in many reported photoinitiating systems,^[^ ^20^, ^21^ ^]^ and its λ_max_ is 247 nm, which is close to an absorption peak of BTXI, BTXI‐Br and BTXIO at 256 , 255  and 242 nm, respectively (see Figure S1a). Additionally, for EDB, its λ_max_ is 310 nm.

**Table 1:** The photochemical properties of BTXI, BTXI‐Br and BTXIO.

	ε_405 nm_ (M^−1^ cm^−1^)	λ_max_ (nm)	ε_max_ (M^−1^ cm^−1^)	E_S1_ (eV)	K^SV^_Iod_ (M^−1^)	K^SV^_EDB_ (M^−1^)	Φ_Iod_ ^a)^	Φ_EDB_ ^b)^	*t*_0_ (ns)	E_ox_ (V)^b)^	E_red_ (V)^b)^
BTXI	5600	457	26 200	2.48	22	26	0.16	0.19	7.5	1.30	−1.16
BTXI‐Br	9100	470	21 000	2.54	21	52	0.16	0.32	6.4	1.40	−1.12
BTXIO	6300	397	7900	3.00	17	20	0.13	0.15	1.5	1.07	−0.71

^a)^ [Iod] = 0.0090 M.

^b)^ [EDB] = 0.0090 M.

^b)^ All potentials in V versus Ag/AgCl. Measurements were performed in DCM under N_2_.

As shown in Figure 1c, the fluorescence emission wavelength of BTXI, BTXI‐Br and BTXIO is 516 , 503 and 445 nm, respectively. A blueshift of the fluorescence emission peak can be significantly observed on BTXIO compared to BTXI, which can be explained by the lower conjugation in molecular structure causing the larger energy gap that leads to a shorter emission wavelength of BTXIO. It is also interesting to observe that the emission intensity of BTXIO is significantly lower than that of BTXI and BTXI‐Br, which could be attributed to the presence of the sulfone group reducing the molecular conjugation. In addition, the lifetimes of these three PCs were also estimated (see Figure S2), and their lifetimes are 7.5 , 6.4, and 1.5 ns, respectively. The obvious difference between BTXI and BTXIO is probably caused by the weakened conjugation in molecular structure as well. The singlet‐state energy (E_S1_) of BTXI, BTXI‐Br and BTXIO, as shown in Figure S3 and Table 1, are 2.48, 2.54, and 3.00 eV, respectively.

**Figure 2:**
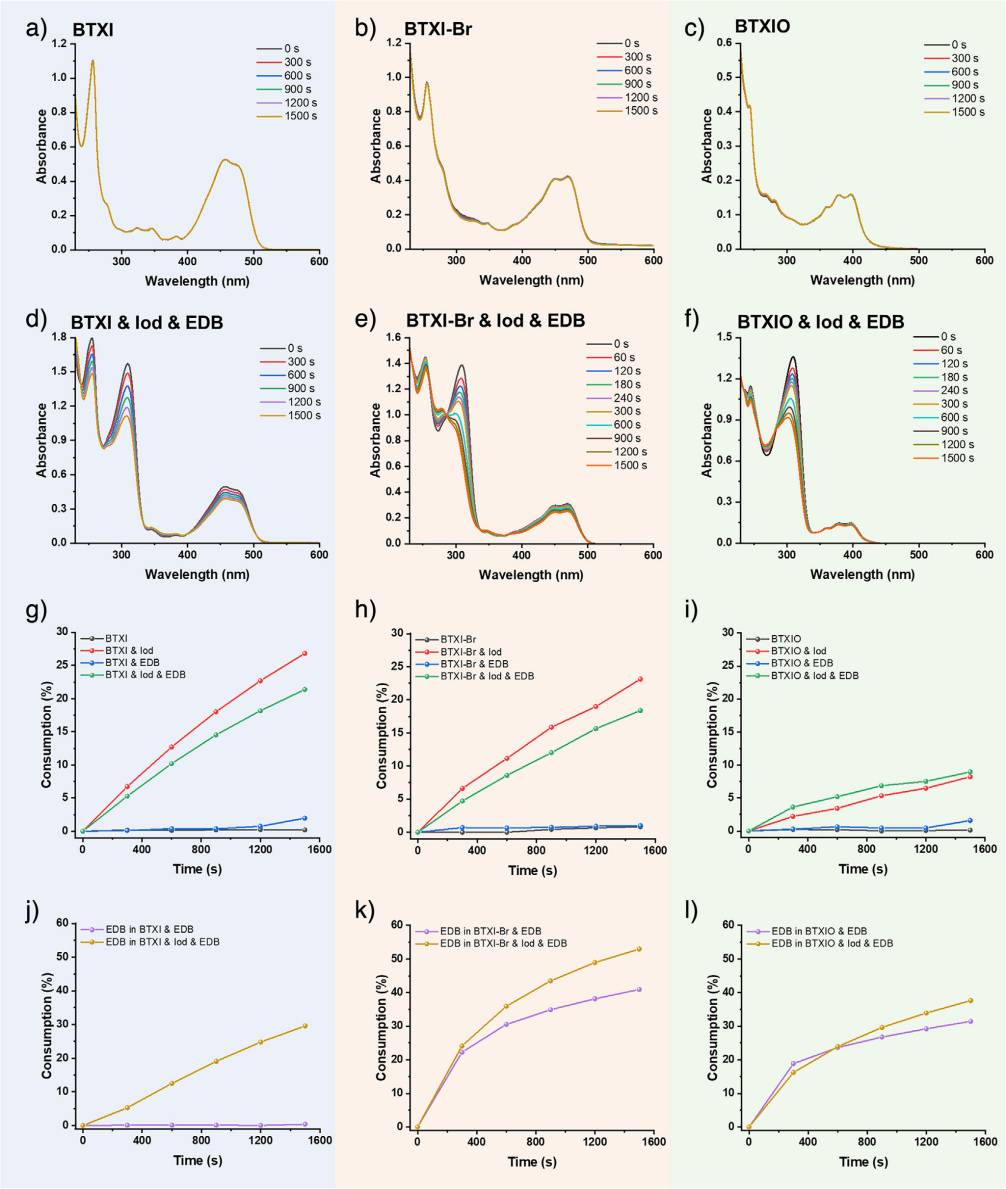
Photolysis of (a) BTXI, b) BTXI‐Br and (c) BTXIO. Photolysis of different three‐component systems: d) BTXI/Iod/EDB, e) BTXI‐Br/Iod/EDB and (f) BTXIO/Iod/EDB. The consumption of PC in different systems: g) BTXI, h) BTXI‐Br, and (i) BTXIO. The consumption of EDB in different systems: j) BTXI, k) BTXI‐Br and (l) BTXIO. The concentration of these systems was controlled at: PC 2 × 10^−5^ M, Iod 4 × 10^−5^ M, EDB 4 × 10^−5^ M in DCM. The light source was LED@405 nm (110 mW cm^−2^).

When these dyes were used as PCs, steady‐state photolysis experiments under the irradiation of LED@405 nm were performed to estimate the photochemical interaction of PC/Iod and PC/EDB. As shown in Figure 2, the consumption of BTXI, BTXI‐Br and BTXIO are all close to 0% upon LED@405 nm exposure for 1500 s, which indicates that all of these PCs present their stability without an obvious photolysis. According to their photostability and good fluorescent performance, this suggests their potential to be incorporated into polymers for the preparation of fluorescent‐coloured materials, as exemplified commercially developed products such as *N*‐octadecyl‐benzok[k,1]thioxanthene‐3,4‐dicarboximide, commercially available as C.I. Solvent Yellow 98 (HOSTASOL YELLOW 3G).^[^ ^45^ ^]^ For the two‐component system of BTXI/Iod, an obvious decline of the absorption peak at ca. 451  and 470 nm, and the consumption of BTXI reaches up to 26.8% after 1500 s, which demonstrates the efficient interaction between BTXI and Iod. Similar results can be clearly observed on BTXI‐Br/Iod and BTXIO/Iod, and their consumptions are 23.1% and 8.2%, respectively. For the PC/EDB systems, the absorption peak of EDB at 310 nm shows a significant decline in BTXI‐Br/EDB and BTXIO/EDB (see Figure S4), and the consumption of EDB in these two systems (calculated from the decrease of the absorbance @310 nm) is 40.9% and 31.4%, respectively. The consumption of EDB in BTXI/EDB is close to 0% under the irradiation of LED@405 nm after 1500 s, implying a poor activity in photocatalytic process. After that, the photolysis of the three‐component systems (PC/EDB/Iod) was also estimated (see Figure 2). An obvious increase of the consumption of EDB while a decrease of PC could be observed in three‐component system compared to corresponding two‐component systems, which implies the catalytic process triggered by PCs between Iod and EDB. All the results suggest that these PCs show excellent potential in multi‐component photoinitiating systems.

In order to further estimate the interaction between PC with Iod or EDB, fluorescence quenching experiments were performed (see Figure S5). As shown in Table 1, the electron‐transfer quantum yields (Φ) are 0.16, 0.16 and 0.13 for BTXI/Iod, BTXI‐Br/Iod, and BTXIO/Iod, while they are 0.19, 0.32, and 0.15 for BTXI/EDB, BTXI‐Br/EDB and BTXIO/EDB, respectively ([Iod] = 0.0090 M, [EDB] = 0.0090 M). When BTXI, BTXI‐Br, and BTXIO worked with Iod, similar electron‐transfer quantum yields were observed.

### Photoinitiating Mechanisms of PC/Iod/EDB

**Figure 3:**
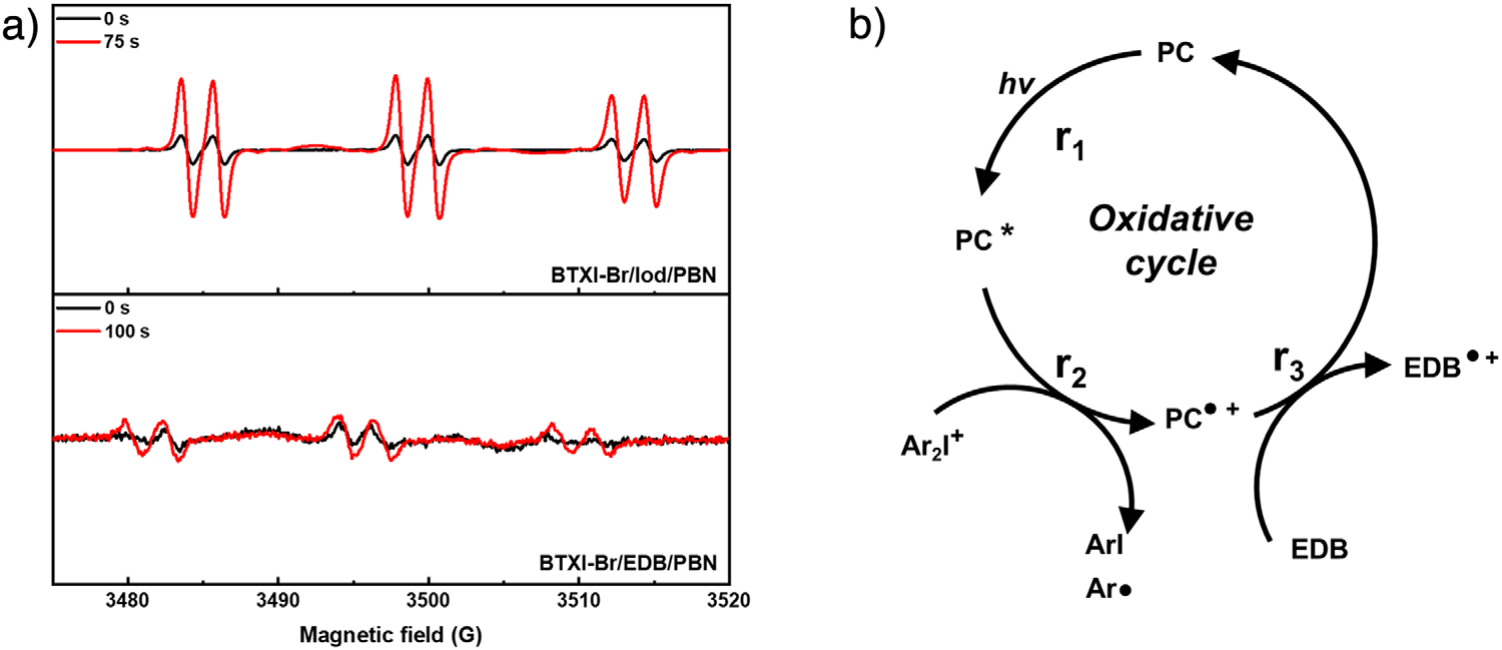
a) ESR of BTXI‐Br/Iod/PBN and BTXI‐Br/EDB/PBN. b) Photochemical mechanism of PC/Iod/EDB.

In order to further study the photoinitiating mechanism of PC/Iod/EDB, ESR experiments were performed on the two‐component PC/Iod and PC/EDB systems in the presence of *N*‐*tert*‐butyl‐*α*‐phenylnitrone (PBN) as a free radical trapping reagent, respectively. As shown in Figure 3a for BTXI‐Br/Iod, the hyperfine splitting constants of PBN/Aryl radical adducts were reported to be a_N _= 14.3 G and a_H _= 2.2 G that characterized the photoproducts formed by the BTXI‐Br/Iod system, namely aryl radicals (Ar^•^).^[^ ^44^ ^]^ For BTXI‐Br/EDB system, the free radical with hyperfine coupling constants a_N _= 14.2 G and a_H _= 2.4 G can be related to the generation of an α‐aminoalkyl radical (EDB^•^).^[^ ^46^ ^]^ However, compared to the intensity of aryl radicals generated in BTXI‐Br/Iod, α‐aminoalkyl radical generated in BTXI‐Br/EDB exhibits a weak intensity, which demonstrates few amounts of α‐aminoalkyl radical could be generated under the irradiation of LED@405 nm after 100 s. Therefore, BTXI‐Br/EDB system may exhibit a poor photoinitiating performance for free radical photopolymerization. In parallel, ESR experiments were performed on other PC/Iod and PC/EDB systems, to further estimate the radical generation from PC/Iod and PC/EDB, respectively. As shown in Figure S6, the intensity of α‐aminoalkyl radical is too low to be observed, which is consistent with the results the steady‐state photolysis of BTXI/EDB, that is, there are almost no degradation happening on BTXI and EDB. As shown in Figure S6, aryl radicals generated from BTXIO/Iod/PBN and BTXI/Iod/PBN both can be observed clearly as well, with a hyperfine splitting constants of a_N _= 14.3 G and a_H _= 2.1 G and a_N _= 14.2 G and a_H _= 2.1 G, respectively.^[^ ^15^ ^]^ Therefore, when PC, Iod and EDB are combined to form a three‐component system, the oxidative reaction from PC/Iod and the reductive reaction from PC/EDB are in competition, and the oxidative reaction from PC/Iod could be the main route. Combined with the results of steady‐state photolysis, in which the addition of EDB could obviously promote the reaction speed, it can be inferred that EDB should be a reductant to complete the photocatalytic cycle in the three‐component systems.

Based on steady‐state photolysis, fluorescence quenching, and ESR experiments, the photochemical mechanism related to PC/Iod/EDB can be proposed (see Figure 3b). Upon light exposure, PC is excited to activated state PC* (r1). An oxidative process occurs subsequently between PC and Iod where the PC acts as an electron donor and Iod serves as an electron acceptor. A single electron transfer between PC and Iod gives rise to the positively charged PC radical cation (PC^•+^) and the aryl radical Ar^•^ (r2).^[^ ^44^ ^]^ With the presence of EDB acting as a reductant, PC is regenerated from PC^•+^ through r3. Therefore, the three‐component system PC/Iod/EDB mainly works through a cycle starting from oxidation step and end with reduction step. Taking BTXI‐Br as an example, as shown in Figure S7 about the CV of BTXI‐Br, the BTXI‐Br radical cation (BTXI‐Br^•+^) can be reduced by EDB, because the reduction potential of BTXI‐Br^•+^ (1.40 V vs. Ag/AgCl) is higher than the oxidation potential of EDB (0.953 V vs. Ag/AgCl). The CV results of BTXI and BTXIO are presented in Figure S7 and Table 1 as well. These results demonstrate that the three‐component system has the potential for a higher initiating efficiency compared to the two‐component system (PC/Iod), a hypothesis that is further verified below.

### Photoinitiating Performance PC/Iod/EDB and 3D Printing Application

**Figure 4:**
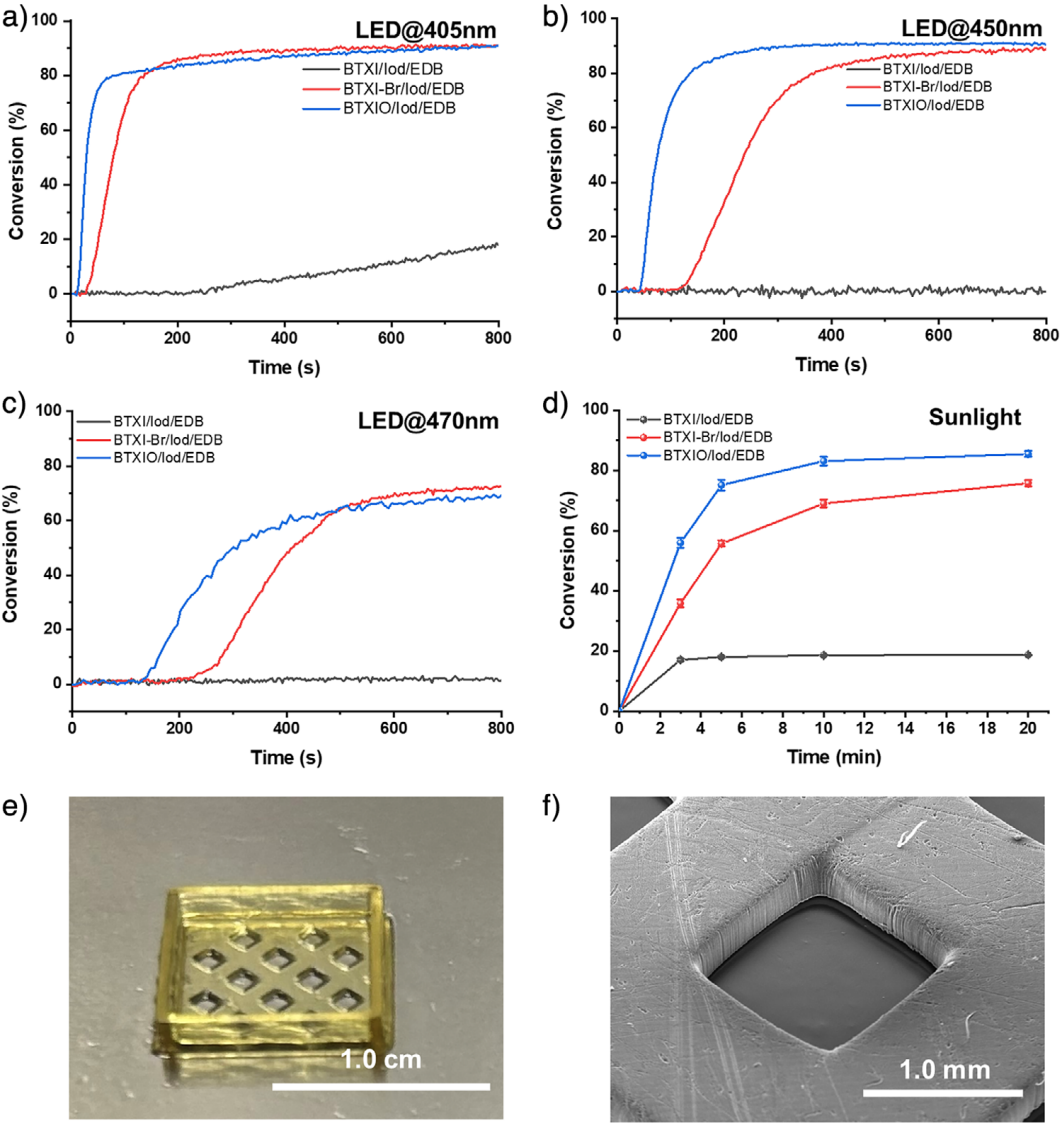
Photopolymerization of 1.4 mm thick PEGDA samples initiated by (a) PC/Iod/EDB under LED@405 nm (110 mW.cm^−2^), b) PC/Iod/EDB under LED@450 nm (70 mW.cm^−2^), c) PC/Iod/EDB under LED@470 nm (45 mW.cm^−2^), d) PC/Iod/EDB under sunlight. The concentration of these systems was controlled at: PC 1 × 10^−6^ mol, Iod 2 × 10^−6^ mol, EDB 2 × 10^−6^ mol in 1 g PEGDA, respectively. For the sunlight‐induced photopolymerization experiments, five parallel experiments were conducted. The results were used to calculate the average values and standard deviations (SD), with the SD values represented as error bars in Figure 4d.

Photopolymerization of poly(ethylene glycol) diacrylate (PEGDA) (Mw ≈ 600) under irradiation of LED@405 nm and LED@450 nm were evaluated by RT‐FTIR, respectively (see Figures 4 and S8). As shown in Figure S8a, Iod/EDB was used as the blank initiating system, and it did not show an effective initiating performance under LED@405 nm irradiation. For PC/EDB‐based systems (see Figure S8b), acrylate function conversions (FCs) of these formulations are 3% (BTXI/EDB), 41% (BTXI‐Br/EDB), and 15% (BEXIO/EDB), respectively, when *t* = 800 s. The high FC of BTXI‐Br/EDB‐based formulation could be related to its more efficient fluorescence quenching performance between BTXI‐Br and EDB, which results in the higher electron‐transfer quantum yield than the two‐component systems BTXI/EDB and BTXIO/EDB. In parallel, their photopolymerization results are consistent with the corresponding steady‐state photolysis of PC/EDB under the irradiation of LED@405 nm, that is, FC(BTXI‐Br/EDB) > FC(BTXIO/EDB) > FC(BTXI/EDB), for EDB consumption, BTXI‐Br/EDB > BTXIO/EDB > BTXI/EDB. The FC of BTXI/EDB (3%) is consistent with the steady‐state photolysis results of BTXI/EDB under the irradiation of LED@405 nm, in which their consumptions are both close to 0. As shown in Figure S8c, the photopolymerization of PEGDA is initiated by PC/Iod systems, and the results clearly demonstrate that BTXI‐Br/Iod and BTXIO/Iod have better initiating performance than BTXI/Iod. FCs of BTXI‐Br/Iod and BTXIO/Iod‐based formulations are 85% and 86%, respectively, while FC of BTXI/Iod‐based formulation is only 16%, when *t* = 800 s (see Table S1). The poor initiating performance of BTXI/Iod could be related to the low concentration and its lower molar extinction coefficient at 405 nm (5600 M^−1^.cm^−1^), compared to that of BTXI‐Br (9100 M^−1^.cm^−1^) and BTXIO (6300 M^−1^.cm^−1^). In order to verify whether the concentration of PC/Iod significantly affects the initiating performance, the concentration of PC/Iod was increased from 1.0 × 10^−6^ mol: 2.0 × 10^−6^ mol to 2.0 × 10^−6^ mol: 4.0 × 10^−6^ mol in 1 g PEGDA. As shown in Figure S8c, the FC of PEGDA significantly increases from 16% to 78% (*t* = 800 s), which clearly demonstrates that the concentration of photoinitiating system could affect the photopolymerization.

As shown in Figure 4, compared to the results of the photopolymerization of PEGDA initiated by PC/Iod and PC/EDB, the three‐component system (PC/Iod/EDB) presents a significant improvement in acrylate function conversion and photopolymerization rate (R_p_). When Iod/EDB was used as reference for initiating the photopolymerization of PEGDA, it exhibits a very low FC value of only 0.3% (*t* = 800 s), which can be considered as no photopolymerization occurring. When *t* = 800 s, FCs of BTXI/Iod/EDB, BTXI‐Br/Iod/EDB and BTXIO/Iod/EDB are 19%, 91% and 91%, which are higher than those of their corresponding two‐component systems initiated photopolymerization. For BTXI/Iod/EDB, its initiating performance can be significantly improved, that is, FC increases from 19% to 75%, through increasing their concentration in PEGDA (see Figure S8d). This is consistent with our observation above. In parallel, as shown in Table S1, compared to *R*_p_ of two‐component systems‐based photopolymerization, *R*_p_ of PC/Iod/EDB initiated photopolymerization has an obvious improvement. Taking BTXI‐Br as an example, *R*_p_ of BTXI‐Br/Iod/EDB is 1.3 s^−1^, which is higher than those of BTXI/Iod (0.8 s^−1^) and BTXI/EDB (0.5 s^−1^). In addition, the duration of the inhibition time significantly affects the photoinitiating efficiency. The inhibition time of the three‐component system is significantly shorter than that of the two‐component system (PC/EDB and PC/Iod), which could indicate the higher efficiency of the three‐component system. Therefore, the use of the three‐component system can efficiently shorten the inhibition time of the polymerization reaction of PEGDA, allowing the reaction to reach the plateau phase faster. Such a system can be effectively used in the application requiring rapid initiation of photopolymerization, such as 3D printing, where the inhibition time should be as short as possible.

According to their UV‐visible absorption spectra, BTXI‐Br and BTXIO both show excellent light absorption properties from 400 to 500 nm, that is, high molar extinction coefficients. In general, photopolymerization efficiency is critically dependent on the irradiation wavelength.^[^ ^47^, ^48^, ^49^ ^]^ Therefore, the blue light (i.e., LED@450 nm and LED@470 nm) and green light (i.e., LED@530 nm) mediated photopolymerization of PEGDA was performed as well to evaluate the relationship between the irradiation wavelength and the photopolymerization efficiency. As shown in Figure 4b, BTXIO/Iod/EDB‐based formulation can reach the plateau phase fast. Its FC can be 91%, which is comparable to the results of photopolymerization under LED@405 nm irradiation (FC = 91%) (see Table S2). For BTXI‐Br/Iod/EDB‐based photopolymerization under LED@450 nm, the time required to reach the plateau phase is approximately 400 s, which is twice that of BTXI‐Br/Iod/EDB‐based photopolymerization under LED@405 nm (ca. 200 s). Interestingly, regardless of whether the photopolymerization is mediated under LED@405 nm or LED@450 nm irradiation, their FCs are very close, at 91% and 89%, respectively. As shown in Figure 4c, when LED@470 nm was used, the photoinititaing efficiency of BTXI‐Br/Iod/EDB and BTXIO/Iod/EDB both significantly decrease. When *t* = 800 s, the FC of BTXI‐Br/Iod/EDB and BTXIO/EDB/Iod‐based formulation can be 72% and 69%, respectively. Their inhibition time of two formulations is longer than that under LED@405 nm and LED@450 nm. When LED@530 nm was used as light source, the FC of BTXI‐Br/Iod/EDB can be 11% when *t* = 800 s (see Figure S8e), while no photopolymerization occurred in BTXI/Iod/EDB and BTXIO/Iod/EDB. Therefore, these three‐component systems can be efficient under irradiation LED@405 nm, LED@450 nm and LED@470nm, but not efficient enough with LED@530 nm.

Unlike artificial light source such as LEDs, sunlight has a broader emission spectrum but lower solar intensity.^[^ ^50^ ^]^ As a results, many photoinitiators that perform efficiently under LED irradiation may exhibit poor efficiency under sunlight exposure.^[^ ^51^, ^52^ ^]^ Here, due to the good light absorption performance, these PC/Iod/EDB systems were used to initiate the photopolymerization under sunlight on March 19, 2025, in Mulhouse, France (see Figure S9). As show in Figure 4d, BTXIO/Iod/EDB exhibits the best initiating efficiency, the FC can reach up to 75% (*t* = 5 min), and the final FC is 82% (*t* = 20 min). For BTXI‐Br/Iod/EDB initiated PEGDA, its FC can be 75% (*t* = 20 min). The initiation efficiency under sunlight is essentially comparable to that under artificial light sources, which fully demonstrates the high efficiency of these systems in initiating photopolymerization under low‐intensity visible light irradiation.

According to the photoinitiating performance of PC/Iod/EDB systems, BTXIO/Iod/EDB‐based formulation exhibits good photopolymerization performance under LED@405 nm irradiation, that is, the fast photopolymerization rate, short inhibition time, and high conversion, which demonstrates its potential for 3D printing. Therefore, digital light processing (DLP) 3D printing was performed with BTXIO/Iod/EDB. The irradiation wavelength of light source is 405 nm. As shown in Figure 4e regarding BTXIO/Iod/EDB‐based formulation, a hollow and porous square structure was successfully printed, and the layer thickness was controlled at 0.02 mm. Subsequently, the details of the bottom surface and the side face of the hollow sections were observed using SEM (see Figure 4f), which exhibits a complete morphology with high resolution.

### Application of Three‐Component Systems (PC/Sulf/EDB) in Photopolymerization and Direct Laser Write

As discussed above, the results described therein clearly present the efficiency of PC/Iod/EDB, a three‐component system, for initiating photopolymerization. Therefore, in order to extend their application range, five different sulfonium salts, which have similar structures to the iodonium salt and considered as possessing similar chemical properties, were synthesized according to previous literature reports in two steps: i) oxidation of thianthrene, phenoxathiine, phenothiazine and dibenzothiophene to the corresponding sulfoxides, ii) reaction of the sulfoxides with either various aromatic derivatives/trifluoromethanesulfonic anhydride (compounds TT‐1, TT‐2, POT and DB) or a boronic acid/BF_3_.Et_2_O (compound PT).^[^ ^53^, ^54^, ^55^, ^56^, ^57^, ^58^ ^]^ These sulfonium salts were then combined with PC and EDB to obtain a new three‐component system (i.e., PC/Sulf/EDB) for initiating photopolymerization. The UV‐visible absorption spectra of Sulfs are presented in Figure S10.

**Figure 5:**
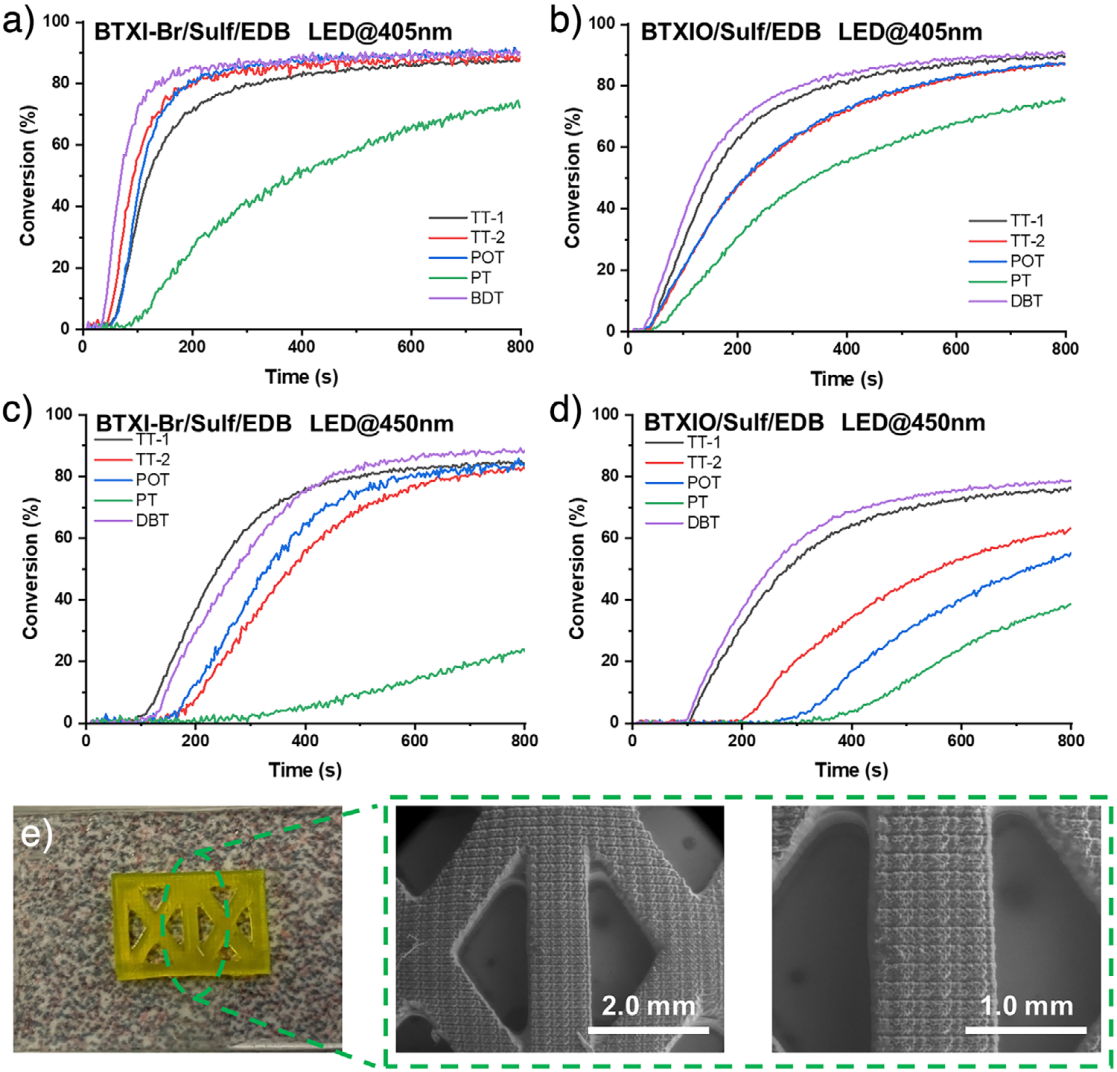
Photopolymerization of 1.4 mm thick PEGDA samples initiated by (a) BTXI‐Br/Sulf/EDB and (b) BTXIO/Sulf/EDB under LED@405 nm (110 mW.cm^−2^), c) BTXI‐Br/Sulf/EDB and (d) BTXIO/Sulf/EDB under LED@450 nm (70 mW.cm^−2^). The concentration of these systems was controlled at: PC 1 × 10^−6^ mol, EDB 2 × 10^−6^ mol, Sulf 2 × 10^−6^ mol in 1 g PEGDA, respectively. For sulfonium salts, they are TT‐1, TT‐2, POT, PT and DBT, respectively. Digital images and SEM images of (e) 3D structures obtained through DLW, containing BTXI‐Br/DBT/EDB. The concentration of these systems was controlled at: PC 1 × 10^−6^ mol, EDB 2 × 10^−6^ mol, Sulf 2 × 10^−6^ mol in 1 g PEGDA, respectively.

As shown in Figure S11 and Table S3, FCs of PC/Sulf‐based formulations are concentrated between ca.75% and ca. 85%, except for BTXI‐Br/PT (FC = 54%). When the three‐component PC/Sulf/EDB system was introduced into the photopolymerization of PEGDA, the photoinitiating performance was significantly improved (see Figure 5a,b). Taking BTXI‐Br/Sulf/EDB as an example, their FCs generally increased to 88%–92%. Even for the less effective BTXI‐Br/PT/EDB system, the final conversion rose from 54% (BTXI‐Br/Sulf) to 76%. Besides, their polymerization rates, R_p_s also increased from ca. 0.4 s^−1^ (BTXI‐Br/Sulf) to 1.5 s^−1^ (BTXI‐Br/Sulf/EDB). The shortened inhibition time and the time to reach the plateau phase indicate a higher polymerization efficiency for the three‐component system. The same improvement can be observed in BTXO/Sulf and BTXIO/Sulf/EDB systems. In addition, the photopolymerization of PEGDA under irradiation of LED@450 nm was performed as well (see Figure 5c,d). Their best FC also can be 89% through using BTXI‐Br/DBT/EDB (see Table S4).

According to their good photoinitiating performance, steady‐state photolysis was performed in PC/Sulf and PC/Sulf/EDB systems, respectively, as well (see Figures S12–S15). Due to the best photoinitiating performance of BTXI‐Br/DBT/EDB under LED@405 nm, DBT was chosen as a model to perform fluorescence quenching in BTXI‐Br/DBT system (see Figure S16 and Table S5). All results demonstrate the efficient interaction among PC, Sulf and EDB.

Among PC/Sulf/EDB systems, the BTXI‐Br/DBT/EDB combination exhibits the best photoinitiating performance under LED irradiation at 405 nm, including the shortest inhibition time and high conversion. Therefore, direct laser write (DLW) experiment was performed BTXI‐Br/DBT/EDB. The irradiation wavelength of light sources is LED@405 nm. As shown in Figure 5e, a 3D pattern was printed through DLW. The good resolution and the route of laser write can be clearly detected from SEM images.

## Conclusion

In this work, we successfully developed three different *N*‐(hexyl)benzothioxanthene‐3,4‐dicarboximides, BTXI, BTXI‐Br and BTXIO as organic PCs with excellent optical stability under visible light irradiation, combined with different compounds, including Iod, EDB and Sulfs to form three‐component systems: PC/Iod/EDB and PC/Sulf/EDB, the former shows better performance both in photopolymerization and application in 3D printing, when the concentration of PC was at 2 × 10^−6^ mol.g^−1^ in PEGDA. These systems exhibit good photoinitiating performance in free radical photopolymerization at LED@405 nm and LED@450 nm. Moreover, these systems can be applied to 3D printing and direct laser writing, and a series of high‐resolution structures were observed clearly. This study provides a new insight for the design and synthesis of novel efficient organo‐photoredox catalysts and the exploration of their application in 3D printing.

## Supporting Information

The authors have cited additional references within the Supporting Information.^[^ ^44^, ^53^, ^54^, ^55^, ^56^, ^57^, ^58^, ^59^, ^60^, ^61^, ^62^, ^63^, ^64^ ^]^

## Conflict of Interests

The authors declare no conflict of interest.

## Supporting information



Supporting Information

## Data Availability

The data that support the findings of this study are available from the corresponding author upon reasonable request.
