# Supplementary material for: Photoredox Catalysts Based on N‐(Hexyl)benzothioxanthene‐3,4‐dicarboximide for Photopolymerization and 3D Printing Under Visible Light
Source: Angew Chem Int Ed Engl. 2025 Apr 25;64(30):e202501442. doi: 10.1002/anie.202501442 (PMC12281092; doi:10.1002/anie.202501442)
Supplement: Supplementary file 1 — Supporting Information [file ANIE-64-e202501442-s001.docx]

Photoredox Catalysts Based on *N*-(Hexyl)benzothioxanthene-3,4-dicarboximide for Photopolymerization and 3D Printing under Visible Light

Bin Song,^[a,b]^ Yijun Zhang,*^[a,b]^ Zheng Liu,^[c]^ Pierre Boulay,^[a,b]^ Céline Dietlin,^[a,b]^ Fabrice Morlet-Savary,^[a,b]^ Michael Schmitt,^[a,b]^ Didier Gigmes,^[c]^ Jean-Michel Becht,*^[a,b]^ Frédéric Dumur,*^[c]^ Jacques Lalevée,*^[a,b]^

[a] Bin Song, Dr. Yijun Zhang, Pierre Boulay, Céline Dietlin, Dr. Fabrice Morlet-Savary, Dr. Michael Schmitt, Dr. Jean-Michel Becht, Prof. Dr. Jacques Lalevée
Université de Haute Alsace, CNRS, IS2M, UMR 7361
F-68100 Mulhouse, France
E-mail: yijun.zhang@uha.fr, jean-michel-becht@uha.fr, jacques.lalevee@uha.fr

[b] Bin Song, Dr. Yijun Zhang, Pierre Boulay, Dr. Céline Dietlin, Dr. Fabrice Morlet-Savary, Dr. Michael Schmitt, Dr. Jean-Michel Becht, Prof. Dr. Jacques Lalevée
Université de Strasbourg

[c] Dr. Zheng Liu, Dr. Didier Gigmes, Dr. Frédéric Dumur
Aix Marseille Univ, CNRS, ICR, UMR 7273
F-13397 Marseille, France

E-mail: frederic.dumur@univ-amu.fr

[*] Corresponding Authors

Dr. Yijun Zhang ; Prof. Dr. Jean-Michel Becht ; Dr. Frédéric Dumur ; Prof. Dr. Jacques Lalevée

Bin Song and Yijun Zhang equally contributed to this work

Materials and Methods

Chemical Compounds

Poly(ethylene glycol) diacrylate (PEGDA) (Mw≈600, SR610) used for free radical photopolymerization was purchased from Sartomer, and the storage inhibitor was not removed. The iodonium salt bis(4-tert-butylphenyl)iodonium hexafluorophosphate (Iod) and ethyl dimethylaminobenzoate (EDB) were purchased from Sartomer Lambson (United Kingdom). The solvent dichloromethane (DCM) was purchased from Sigma-Aldrich and used for UV-visible absorption, photolysis and fluorescence experiments. *N*-tert-butyl-α-phenylnitrone (PBN) was obtained from TCI Europe (Paris, France) and utilized as the free radical trapping agent. Tert-butylbenzene was purchased from TCI Europe used as the solvent in ESR-ST experiments. LED lamps (LED@405nm, LED@450nm, LED@470nm, LED@530nm) were purchased from THORLABS, and their emission spectra are presented in Figure S1.

PC Synthesis

BTXI and BTXI-Br were synthesized as previously reported in the literature, without modification and in similar yields.^[1]^ Starting materials and additives were all from Sigma Aldrich (Europe). High resolution mass spectra were obtained from Bruker matrix assisted laser desorption ionization time-of-flight tandem mass spectrometry (MALDI-TOF-TOF).

**Synthesis of 2-hexyl-1H-thioxantheno[2,1,9-def]isoquinoline-1,3(2H)-dione 6,6-dioxide (BTXIO)**

*m*-Chloroperbenzoic acid (mCPBA) (1.78 g, 10.29 mmol, M = 172.56 g/mol) was added to a stirred solution of 2-hexyl-1H-thioxantheno[2,1,9-def]isoquinoline-1,3(2H)-dione 6,6-dioxide (1.20 g, 2.57 mmol, M = 466.39 g/mol). The mixture was stirred overnight at room temperature and the solution was quenched with a saturated solution of Na_2_S_2_O_3_. The organic phase was extracted with methylene chloride and washed with a saturated solution of NaHCO_3_. The organic phase was dried over MgSO_4_ and concentrated under vacuum. After evaporation of the volatiles, the raw material was recrystallized in a dichloromethane/ether mixture, furnishing an orange powder (0.31g, yield 95%).

**^1^H NMR** (400 MHz, CDCl_3_) δ 8.91 (s, 1H), 8.75 (d, *J* = 8.0 Hz, 1H), 8.62 (d, *J* = 8.1 Hz, 1H), 8.34 – 8.25 (m, 2H), 7.84 – 7.71 (m, 2H), 4.20 – 4.14 (m, 2H), 1.73 (q, *J* = 7.7 Hz, 2H), 1.45 – 1.27 (m, 6H), 0.92 – 0.88 (m, 3H).

**^1^H NMR** (400 MHz, CDCl_3_): 5-bromo-2-hexyl-1*H*-thioxantheno[2,1,9-*def*]isoquinoline-1,3(2*H*)-dione 6,6-dioxide

High resolution mass spectra

HRMS of BTXI [M]^+^: calcd for C_24_H_21_NO_2_S 387.1293, found 387.1291.

HRMS of BTXI-Br [M]^+^: calcd for C_24_H_20_BrNO_2_S 465.0398, found 465.0390.

HRMS of BTXIO [M]^+^: calcd for C_24_H_21_NO_4_S 419.1191, found 419.2961.

Synthesis of Sulfonium Salts

**General information**

High resolution mass spectra were obtained from Bruker Compass DataAnalysis 5.3 (ESI as ion resource).

**General procedure A for the synthesis of sulfoxides (for TT, PT and DBT).**

A round-bottom flask was charged with either thianthrene, phenoxathiine or dibenzothiophene (1.0 equiv.) in a 75 : 25 mixture of acetonitrile / H_2_O. The mixture is stirred at rt and if necessary slightly heated to dissolve the starting materials. Cerium ammonium nitrate (CAN, 3.0 equiv.) was then added in small portions at rt. The reaction mixture was then stirred at rt and the progress of the reaction was monitored by TLC analysis (using as eluent a 8 : 2 mixture of cyclohexane / EtOAc). At the end of the reaction, H_2_O was added to the reaction mixture. The aqueous layer was extracted 3 times with Et_2_O. The organic layers were combined, dried with anhydrous MgSO_4_ and the solvent was evaporated under reduced pressure. The crude product was purified by flash-chromatography on silica gel using as eluent a 8 : 2 mixture of cyclohexane / EtOAc to afford after drying under vacuum (0.1 mbar) the sulfoxides.^[2]^

**Thianthrene-5-oxide** was obtained starting from thianthrene (1.73 g, 8.0 mmol), CAN (13.2 g, 24 mmol) in acetonitrile / H_2_O (160 mL). Sulfoxide of TT was obtained as a white solid (1.76 g, 7.6 mmol, 95 %). The spectroscopic data are in agreement with a previous literature report.^[3]^

**^1^H NMR** (400 MHz, CDCl_3_) δ (ppm) 7.94 (ddd, J = 7.6, 1.2, 0.4 Hz, 2H), 7.65 (ddd, J = 7.6, 1.2, 0.4 Hz, 2H), 7.57 (td, J = 7.6, 1.3 Hz, 2H), 7.45 (td, J = 7.6, 1.3 Hz, 2H).

**^1^H NMR** (CDCl_3_, 300 MHz): Thianthrene-sulfoxide


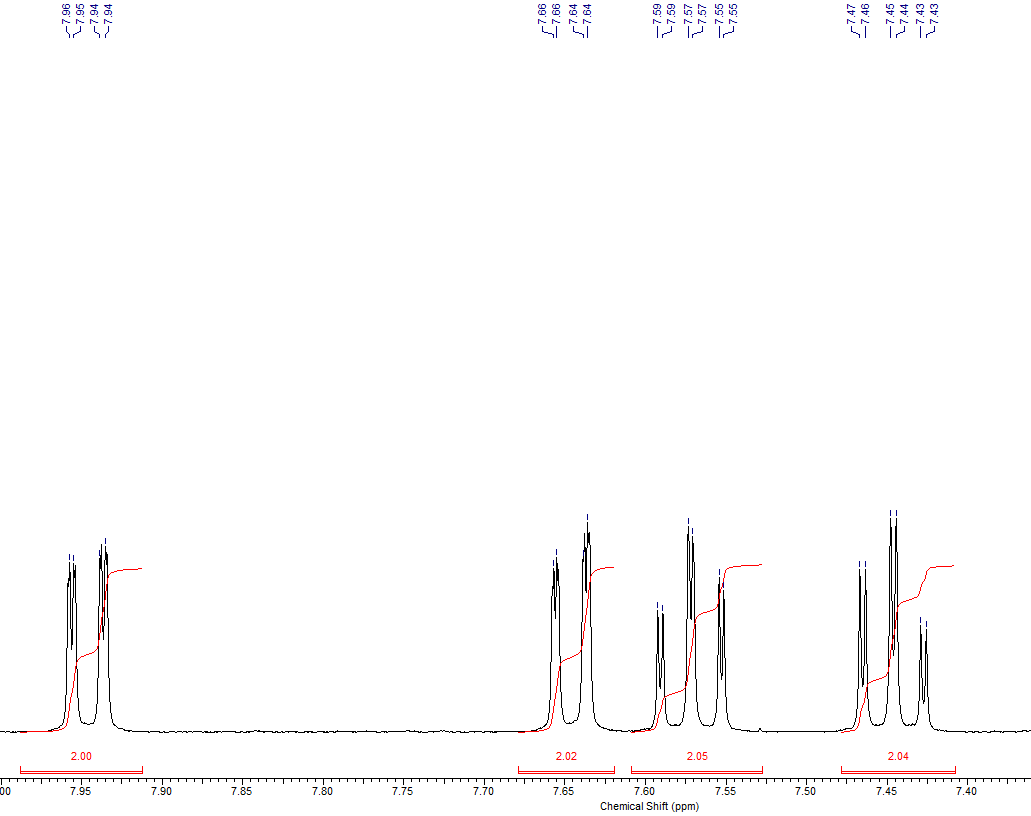


**Phenoxathiine-10-oxide** was obtained starting from phenoxathiine (1.0 g, 5.0 mmol), CAN (8.22 g, 15 mmol) in acetonitrile / H2O (100 mL). Sulfoxide of POT was obtained as a white solid (1.07 g, 4.9 mmol, 99 %).

**^1^H NMR** (300 MHz, CDCl_3_) δ (ppm) 7.95 (dd, J = 8.1, 1.5 Hz, 2H), 7.73-7.62 (m, 2H), 7.48-7.38 (m, 4H). The spectroscopic data are in agreement with a previous literature report.^[3]^

**^1^H NMR** (CDCl_3_, 300 MHz): Phenoxathiine-10-oxide


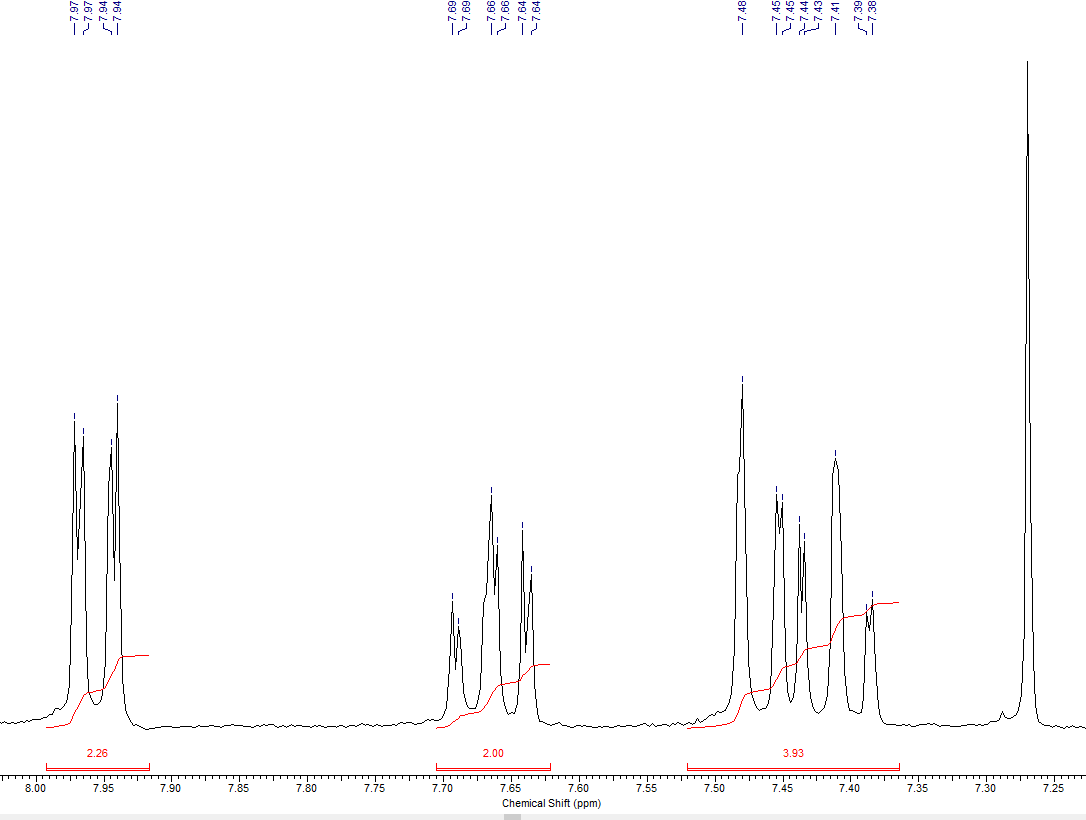


**Dibenzothiophene-9-oxide** was obtained starting from dibenzothiophene (184 mg, 1.0 mmol), CAN (1.64 g, 3.0 mmol) in acetonitrile / H_2_O (20 mL). Sulfoxide of DBT was obtained (176 mg, 0.9 mmol, 88 %) as a yellowish solid. The spectroscopic data are in agreement with a previous literature report.^[3]^

**^1^H NMR** (500 MHz, CDCl_3_) δ (ppm) 8.00 (d, J = 7.5 Hz, 2H), 7.84 (d, J = 7.5 Hz, 2H), 7.62 (td, J = 7.5, 1.0 Hz, 2H), 7.52 (td, J = 7.5, 1.0 Hz, 2H). HRMS

**^1^H NMR** (CDCl_3_, 300 MHz): Dibenzothiophene-9-oxide


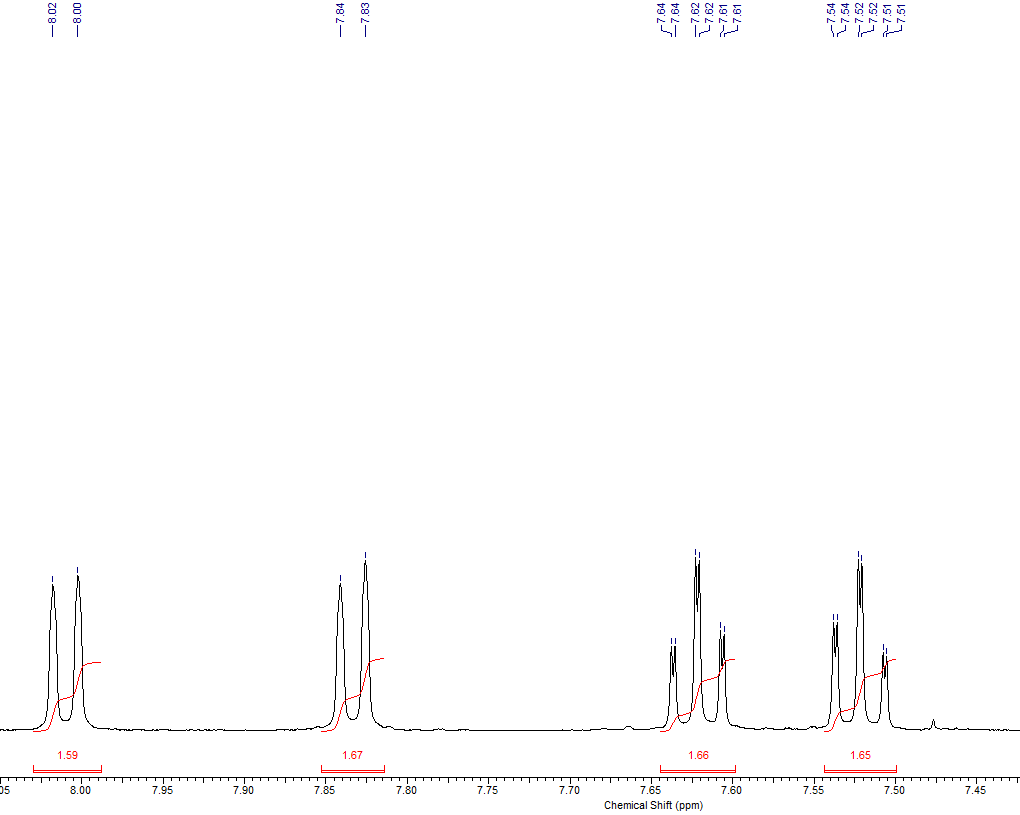


**General procedure B for the synthesis of sulfoxide (PT).**

**10-Methylphenothiazine 5-oxide.** Step 1.^[4-6]^ A round-bottom flask was charged with phenothiazine (997 mg, 5.0 mmol, 1.0 equiv.) in dry DMF (10 mL). Then, MeI (0.34 mL, 5.5 mmol, 1.1 equiv.) and NaH (60% in oil dispersion: 420 mg, 12.5 mmol, 1.5 eq.) were successively and slowly added at rt. The reaction mixture was stirred at rt for 2 h. The reaction mixture was then quenched by addition of H_2_O (15 mL). The aqueous layer was extracted 3 times with Et_2_O (3x 20 mL). The organic layers were combined, dried with anhydrous MgSO_4_ and finally concentrated under reduced pressure. The crude product was dried under vacuum (0.1 mbar) and used for the next step without further purification (purity of ca. 99 % by ^1^H-NMR). Step 2. The crude product obtained after step 1 was dissolved in AcOH (12.5 mL). Then, a solution of NaNO_2_ (434 mg, 6.2 mmol, 1.25 equiv.) in H_2_O (1 mL) was added slowly. The reaction mixture was stired at rt for 2 h. After reaction, H_2_O (25 mL) was added to the solution. The aqueous layer was extracted with 3 times with DCM (3x25 mL). The organic layers were combined, washed with an aqueous saturated NaHCO_3_ solution (20 mL), dried over MgSO_4_ and final concentrated under reduced pressure. Sulfoxide of PT was obtained without any further purification as a yellowish solid (792 mg, 3.45 mmol, 69 %). The spectroscopic data are in agreement with a previous literature report.^[5]^

**^1^H NMR** (300 MHz, CDCl_3_) δ (ppm) 7.95 (dd, J = 8.1, 1.2 Hz, 2H), 7.69-7.59 (m, 2H), 7.41 (d, J = 8.7 Hz, 2H), 7.27 (t, J = 8.0 Hz, 2H), 3.79 (s, 3H).

**^1^H NMR** (CDCl_3_, 400 MHz): N-methylphenothiazine sulfoxide


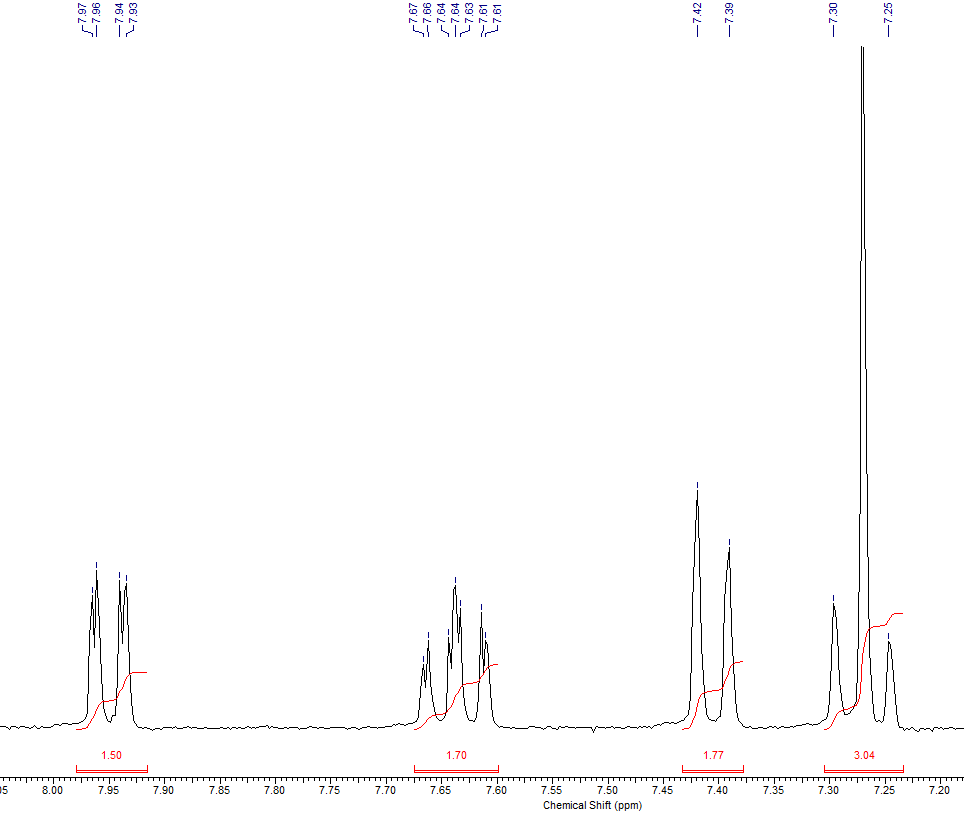


**General procedure D for the synthesis of sulfonium salts (TT-1, TT-2).**

A flame dried round bottom flask under an atmosphere of argon was charged with thianthrene-5-oxide (1.0 equiv.) and the (hetero)aromatic compound (1.0 equiv.) in dry DCM (0.12 M). The mixture was cooled to ca. -40°C. Then, Tf_2_O (1.2 equiv.) was added dropwise at ca. -40 °C. The reaction mixture was stirred at ca. -40°C for 30 min, then warmed to rt. The reaction was monitored by TLC analysis (using as eluent a 10 : 1 mixture of DCM / MeOH). At the end of the reaction, a saturated aqueous NaHCO_3_ solution (25 mL) was added. The aqueous phase was extracted with 3 times with DCM (3x30 mL). The combined organic layers were dried with anhydrous MgSO_4_ and the solvent was evaporated under reduced pressure. The crude product was purified by crystallization from DCM / Et_2_O to afford the desired aryl thianthrenium salts.^[7, 8]^

**5-(4-Methoxyphenyl)-5H-thianthren-5-ium trifluoromethanesulfonate (TT-1)** was obtained starting from sulfoxide of TT (2.32 g, 10 mmol), anisole (1.1 mL, 10 mmol), Tf_2_O (2.0 mL, 12 mmol) in dry DCM (80 mL). Thianthrenium salt was obtained as a white solid (4.25 g, 9.6 mmol, 90 %). The spectroscopic data are in agreement with a previous literature report.^[9]^

**^1^H NMR** (400 MHz, CDCl_3_) δ (ppm) 8.59-8.57 (m, 2H), 7.83-7.72 (m, 6H), 7.32 (d, J = 9.2 Hz, 2H), 6.95 (d, J = 9.2 Hz, 2H), 3.81 (s, 3H). ^19^F NMR (470 MHz, CDCl_3_) δ (ppm) -77.7 (s, 3F).

**^1^H NMR** (CDCl_3_, 500 MHz): 5-(4-methoxyphenyl)-5H-thianthren-5-ium trifluoromethanesulfonate


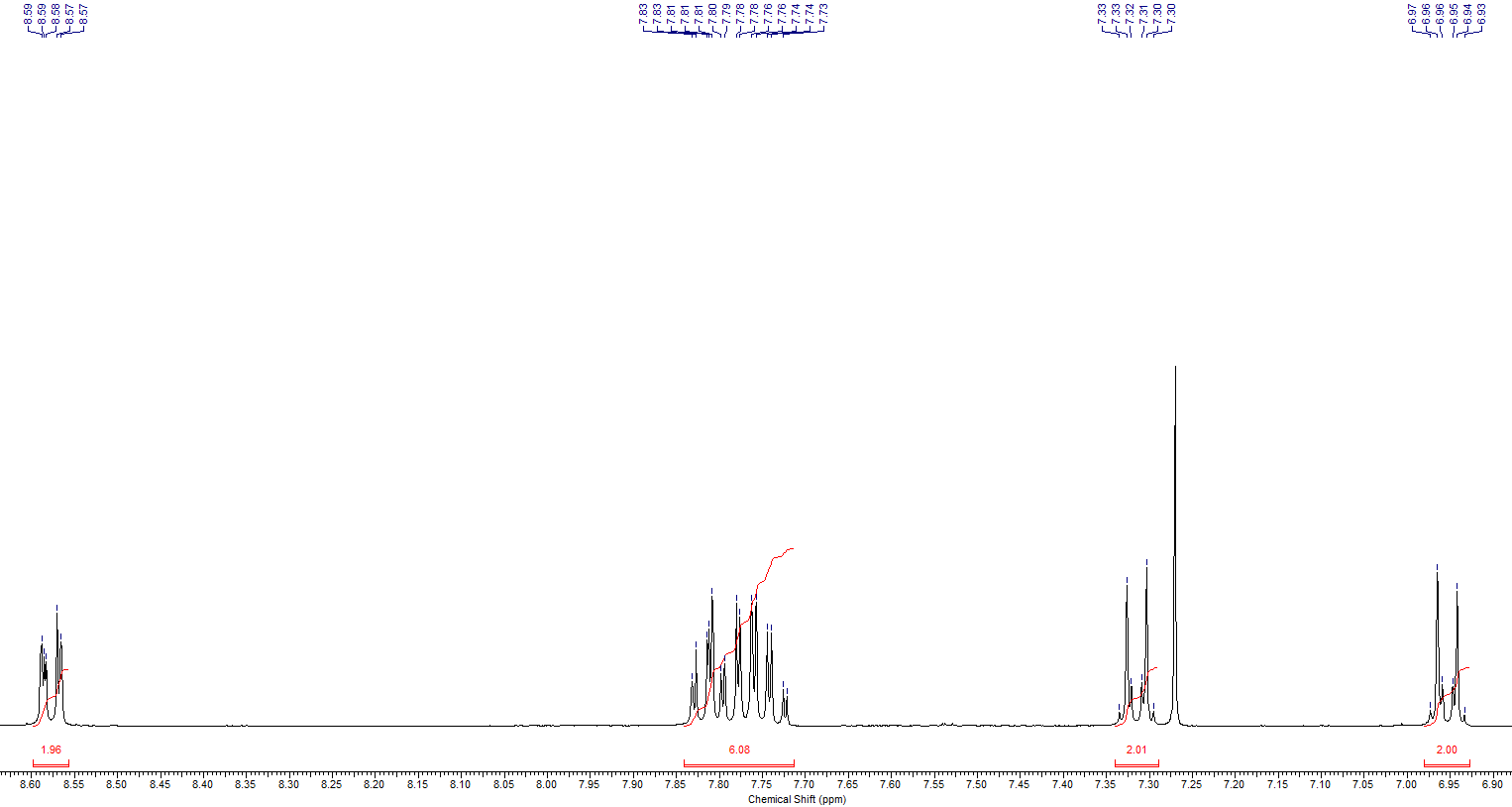


**^19^F NMR** (CDCl_3_, 476 MHz): 5-(4-methoxyphenyl)-5H-thianthren-5-ium trifluoromethanesulfonate

**HRMS of TT-1 [M]^+^:** calcd for [C_19_H_15_OS_2_]^+^ 323.0559, found 323.0558; calcd for [CF_3_O_3_S]^-^ 148.9526, found 148.9526;


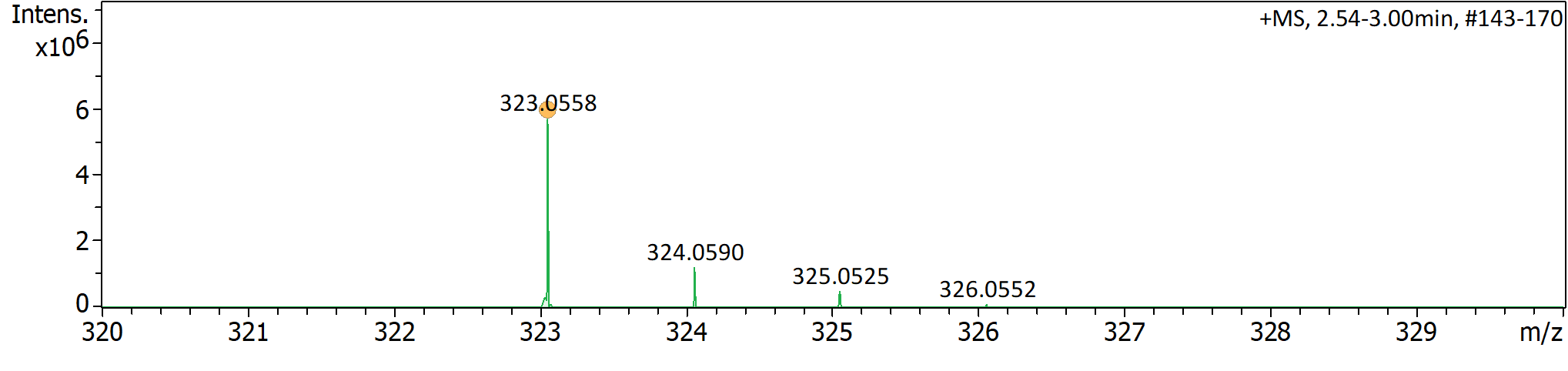


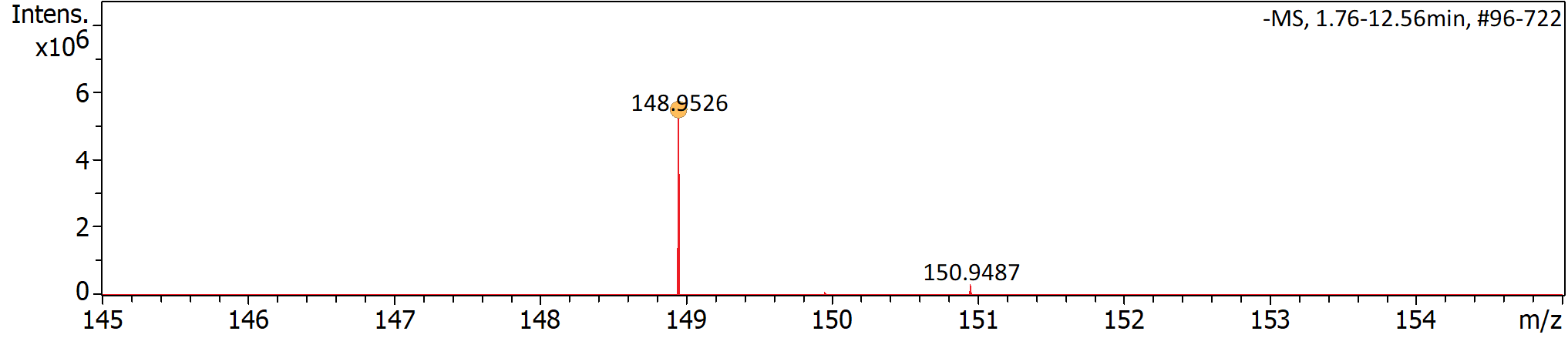


**5-(4-Tert-butylphenyl)-5H-thianthren-5-ium trifluoromethanesulfonate** **(TT-2)** was obtained starting from sulfoxide (2.32 g, 10 mmol), tert-butylbenzene (1.6 mL, 10 mmol), Tf_2_O (2.0 mL, 12 mmol) in dry DCM (80 mL). Thianthrenium salt was obtained as a white solid (4.03 g, 8.1 mmol, 81 %). The spectroscopic data are in agreement with a previous literature report.^[9]^

**^1^H NMR** (400 MHz, CDCl_3_) δ (ppm) 8.69-8.66 (m, 2H), 7.84-7.74 (m, 6H), 7.45 (d, J = 8.8 Hz, 2H), 7.16 (d, J = 8.8 Hz, 2H), 1.26 (s, 9H).

**^1^H NMR** (CDCl_3_, 500 MHz): 5-(4-*tert*-butylphenyl)-5H-thianthren-5-ium trifluoromethanesulfonate


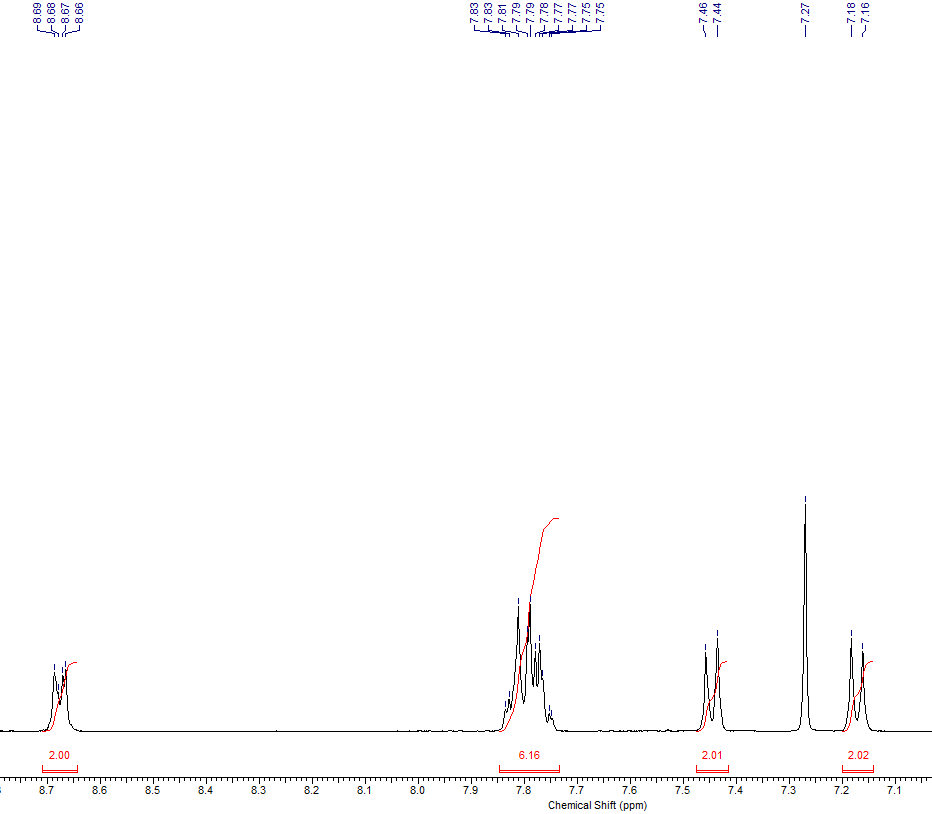


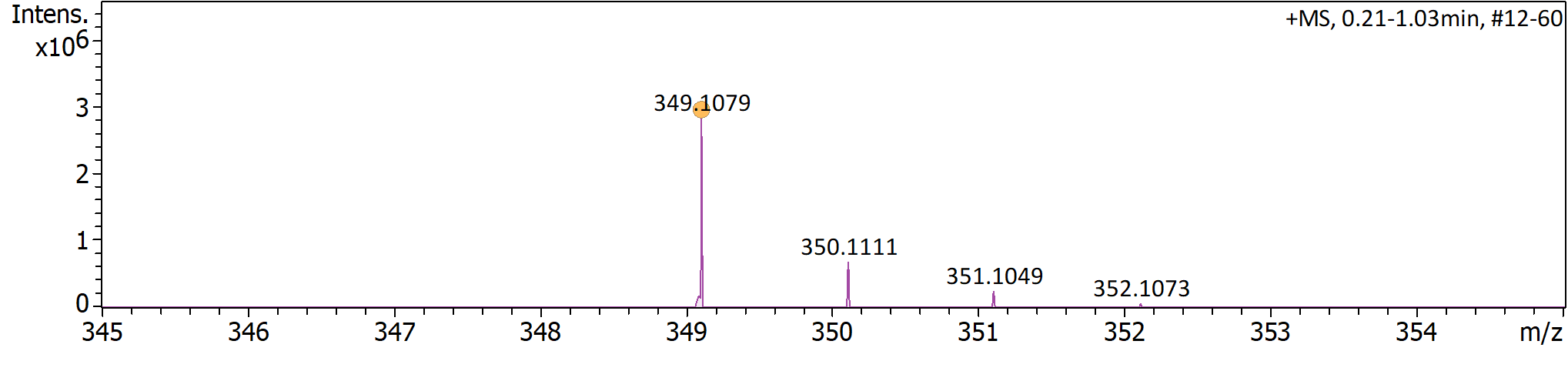
**HRMS of TT-2 [M]^+^:** calcd for [C_22_H_21_S_2_]^+^ 349.1079, found 349.1079; calcd for [CF_3_O_3_S]^-^ 148.9526, found 148.9525.

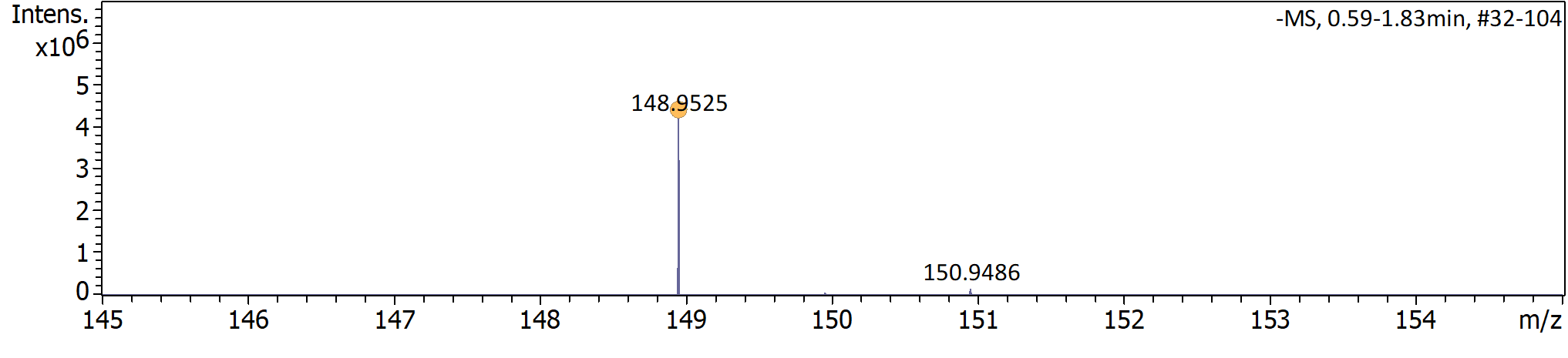

**General procedure E for the synthesis of sulfonium salts (POT):**

**5-(4-Tert-butylphenyl)phenoxathiinium trifluoromethanesulfonate**

A flame dried round bottom flask under an atmosphere of argon was charged with phenoxathiine sulfoxide (1.06 g, 4.9 mmol, 1.0 equiv.) tert-butylbenzene (0.70 mL, 4.9 mmol, 1.0 equiv.) in dry DCM (45 mL). The mixture was cooled to ca. -40°C. Then, Tf_2_O (0.9 mL, 5.3 mmol ,1.2 equiv.) was added dropwise at ca. -40 °C. The reaction mixture was stirred at ca. -40°C for 30 min, then warmed to rt. The reaction was monitored by TLC analysis (using as eluent a 10 : 1 mixture of DCM / MeOH). At the end of the reaction, MeOH was added until a discoloration of the reaction mixture. Then, the mixture was concentrated under reduced pressure. The crude product was crystallized by addition Et_2_O. The solid was then filtered off to afford the phenoxathiinium salt as a white solid (1.60 mg, 0.32 mmol, 75 %). The spectroscopic data are in agreement with a previous literature report. ^[7, 8]^

**^1^H NMR** (400 MHz, CDCl_3_) δ (ppm) 8.26 (dd, J = 8.0, 1.6 Hz, 2H), 7.80 (ddd, J = 8.4, 7.3, 1.6 Hz, 2H), 7.72 (d, J = 8.8 Hz, 2H), 7.58 (dd, J = 8.4, 1.2 Hz, 2H), 7.54-7.50 (m, 4H), 1.26 (s, 9H).

**^1^H NMR** (CDCl_3_, 300 MHz): 5-(4-*tert*-butylphenyl)phenoxathiinium trifluoromethanesulfonate


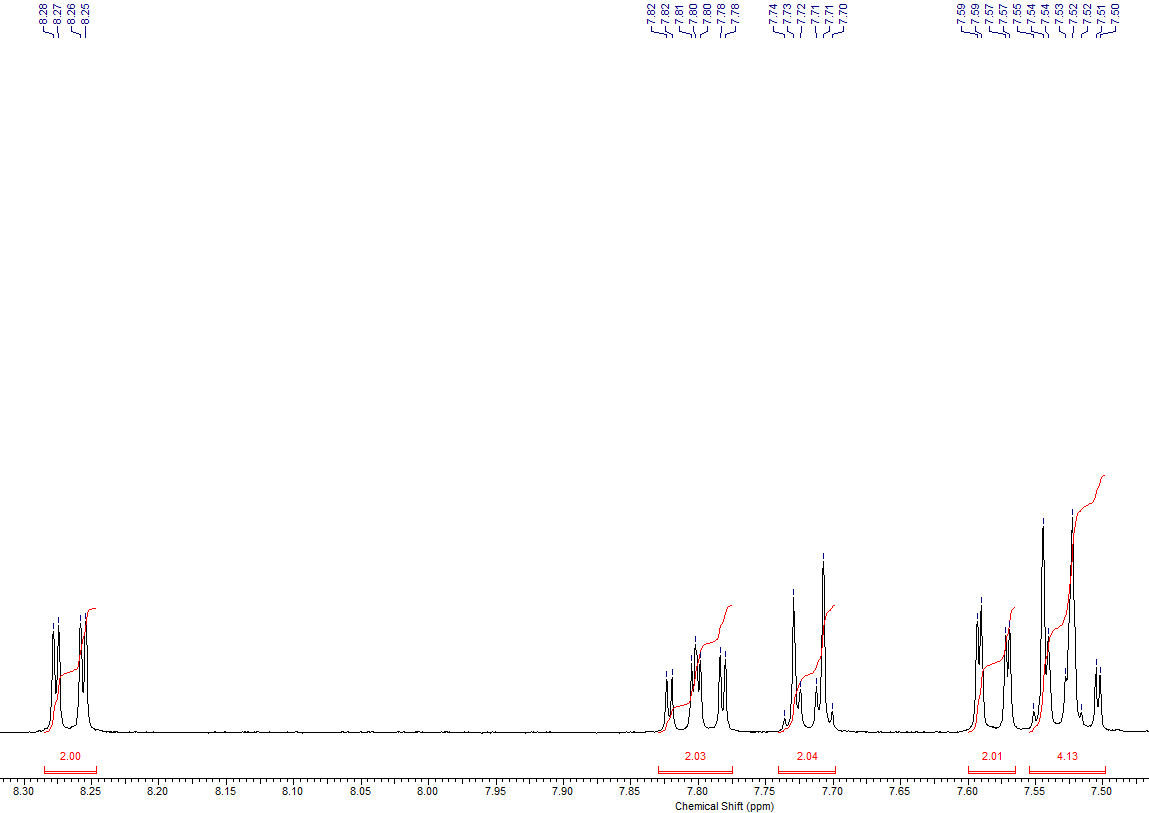


**HRMS of POT [M]^+^:** calcd for [C_22_H_21_OS]^+^ 349.1308, found 333.1307; calcd for [CF_3_O_3_S]^-^ 148.9526, found 148.9528.


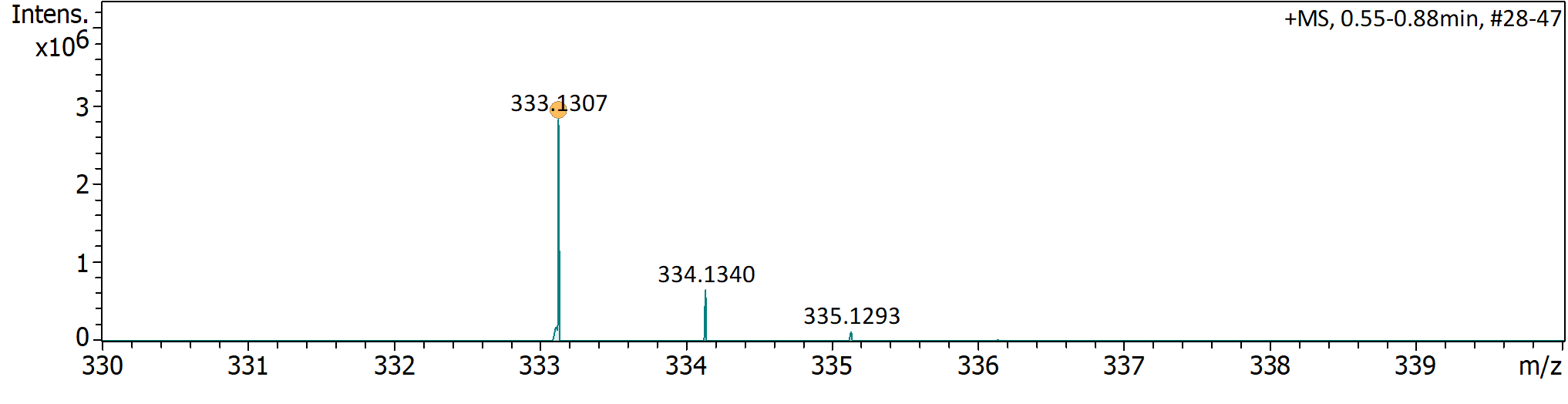


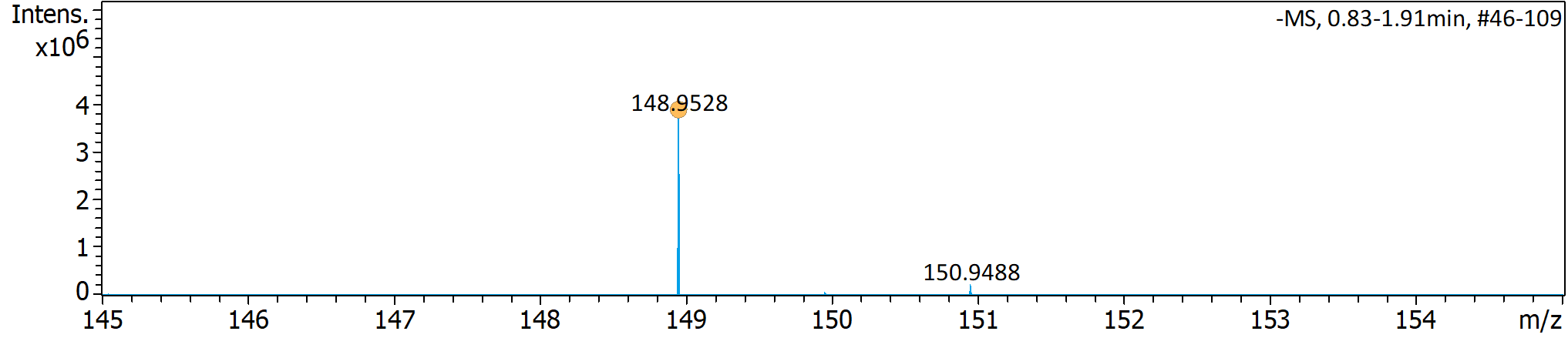


**General procedure F for the synthesis of sulfonium salts (PT)**

**10-Methyl-5-(4-methoxyphenyl)phenothiazinium trifluoromethanesulfonate.** ^[4-6]^

A flame dried round bottom flask under an atmosphere of argon was charged with N-methylphenothiazine sulfoxide (688 mg, 3.0 mmol, 1.0 equiv.), 4-methoxyphenylboronic acid (548 mg, 3.6 mmol, 1.2 equiv.) and BF_3_.OEt_2_ (0.97 mL, 7.8 mmol, 2.6 equiv.) in dry DCM (60 mL). The reaction mixture was stirred at rt for 3 h. Then, the solvent was evaporated under reduced pressure. The obtained solid was dissolved in a mixture of DCM (30 mL) and an aqueous saturated solution of NaBF_4_ (30 mL) was added. The aqueous layer was then directly extracted with 3 times with DCM (3x50 mL). The combined organic layers were dried with anhydrous MgSO4 and the solvent was evaporated under reduced pressure The crude product was washed 2 times with Et_2_O (2x20 mL) and dried under vacuum. N-methylphenothazinium salt was obtained as a beige solid (1.14 g, 2.8 mmol, 90%). The 1H-NMR spectrum is in agreement with a previous literature report.^[5]^

**^1^H NMR** (500 MHz, CDCl_3_) δ (ppm) 8.17 (dd, J = 8.0, 1.5 Hz, 2H), 7.81 (td, J = 8.5, 1.5 Hz, 2H), 7.50 (d, J = 8.5, 2H), 7.45-7.41 (m, 2H), 7.35 (d, J = 9.0 Hz 2H), 6.96 (d, J = 9.0 Hz, 2H), 3.79 (s, 3H), 3.76 (s, 3H). ^19^F NMR (470 MHz, CDCl_3_) δ (ppm) -78.2 (s, 3F).

**^1^H NMR** (CDCl_3_, 500 MHz): 10-methyl-5-(4-methoxyphenyl)phenothiazinium trifluoromethanesulfonate

**^19^F NMR** (CDCl_3_, 470 MHz): 10-methyl-5-(4-methoxyphenyl)phenothiazinium trifluoromethanesulfonate

**HRMS of PT [M]^+^:** calcd for [C_20_H_18_NOS]^+^ 320.1104, found 320.1104; calcd for [BF_4_]^-^ 87.0035, found 87.0036.


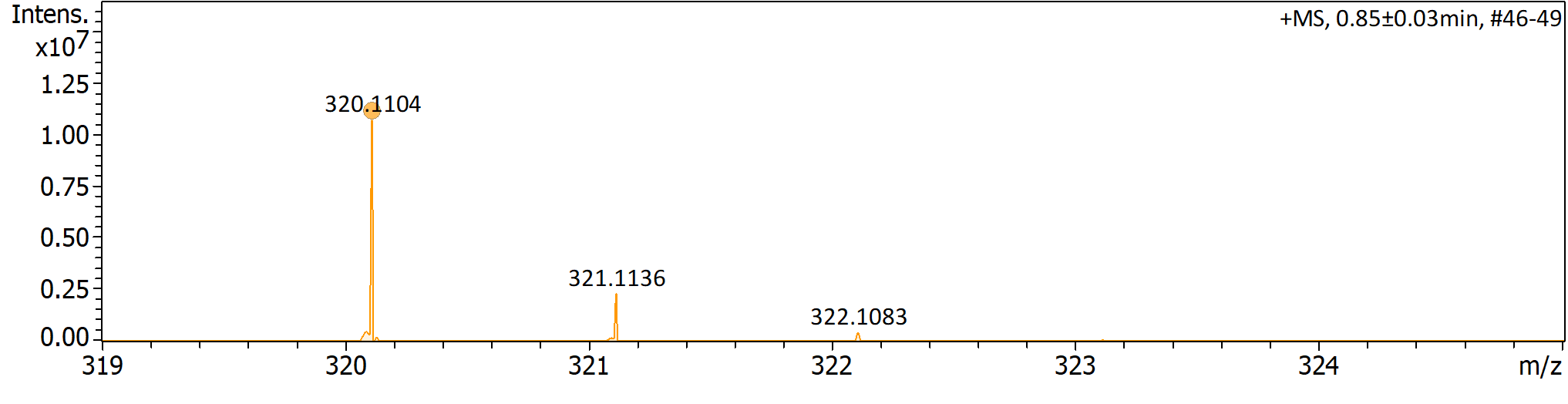


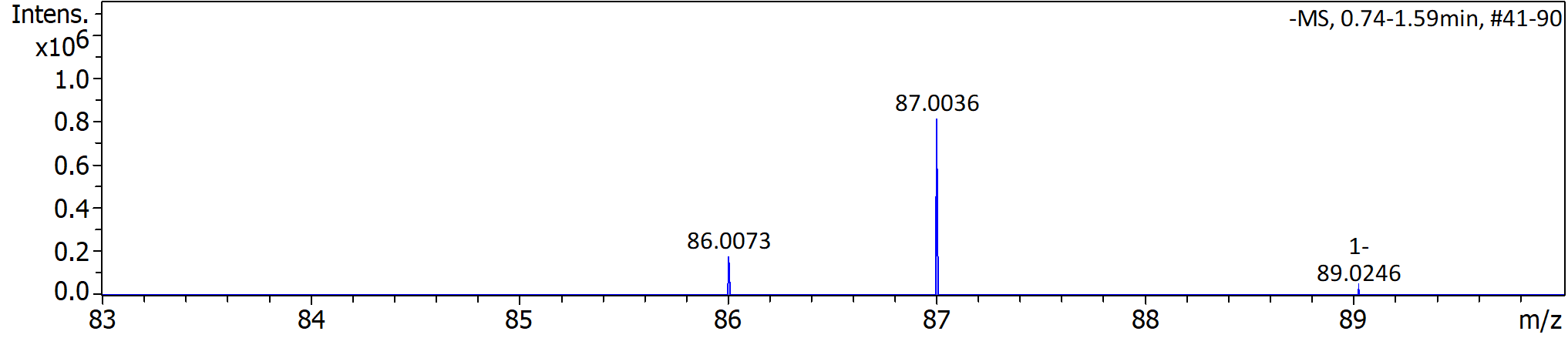

**General procedure F for the synthesis of sulfonium salts (DBT):**

**5-(4-Tert-butylphenyl)-5H-dibenzo[b,d]thiophen-5-ium trifluoromethanesulfonat.** ^[7, 8]^

A flame dried round bottom flask under an atmosphere of argon was charged with dibenzothiophene sulfoxide (1.10 g, 5.5 mmol, 1 equiv.) and tert-butylbenzene (0.78 mL, 5 mmol, 1 equiv.) in dry DCM (50 mL). The mixture was cooled to ca. -40°C. Then, Tf_2_O (1.0 mL, 6 mmol, 1.2 equiv.) was added dropwise at ca. -40 °C. The reaction mixture was stirred at ca. -40°C for 30 min, then warmed to rt. The reaction was monitored by TLC analysis (using as eluent a 10 : 1 mixture of DCM / MeOH). MeOH was added until a discoloration of the reaction mixture. Then, the mixture was concentrated under reduced pressure. The crude product was crystallized by addition Et_2_O. The crude product was purified by cristallisation by addition of DCM / Et_2_O to afford the dibenzothiophenium salt as a white solid (1.85 g, 0.4 mmol, 79 %). The spectroscopic data are in agreement with a previous literature report. ^[7, 8]^

**^1^H NMR** (400 MHz, CDCl_3_) δ (ppm) 8.20 (d, J = 8.0 Hz, 2H), 8.16 (d, J = 7.7 Hz, 2H), 7.87 (t, J = 7.9, 2H), 7.68 (t, J = 7.6 Hz, 2H), 7.62 (d, J = 8.8 Hz, 2H), 7.53 (d, J = 8.8 Hz, 2H), 1.28 (s, 9H).

**^1^H NMR** (CDCl_3_, 500 MHz): 5-(4-*tert*-butylphenyl)-5H-dibenzo[b,d]thiophen-5-ium trifluoromethanesulfonate


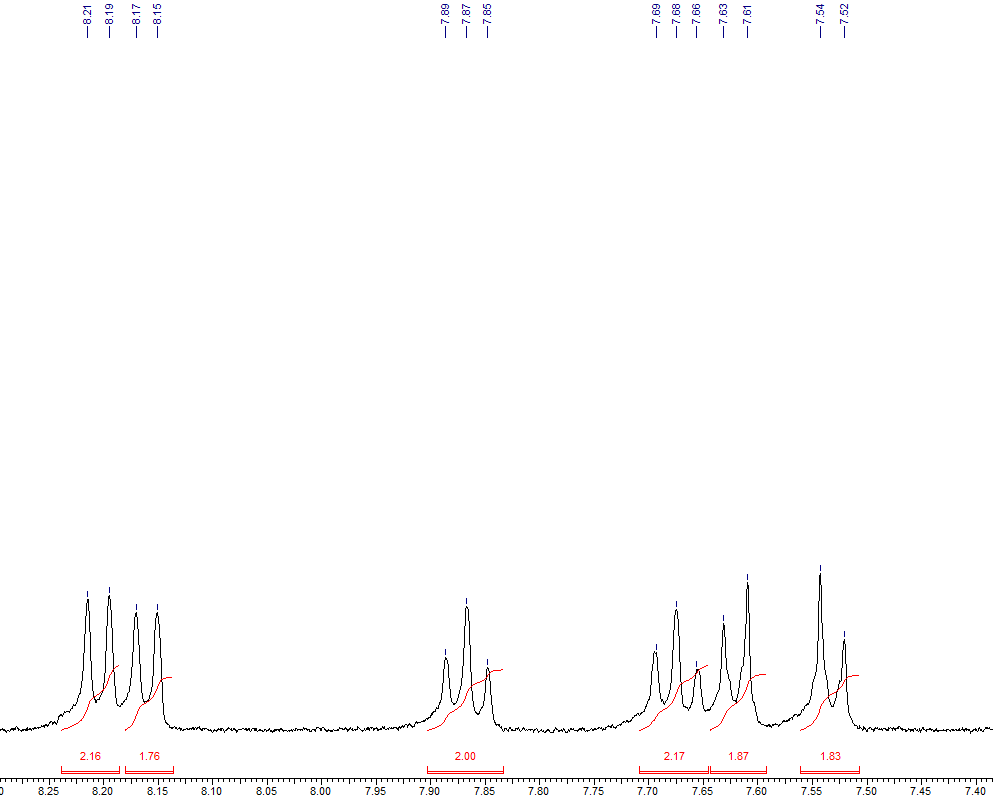


**HRMS of DBT [M]^+^:** calcd for [C_22_H_21_S]^+^ 317.1358, found 317.1357; calcd for [CF_3_O_3_S]^-^ 148.9526, found 148.9528.


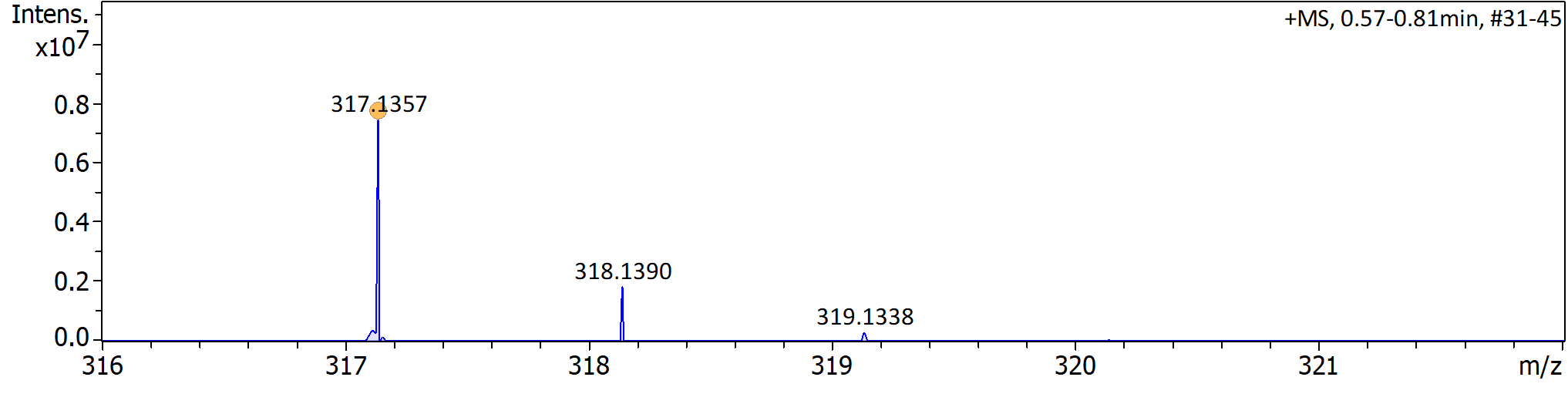


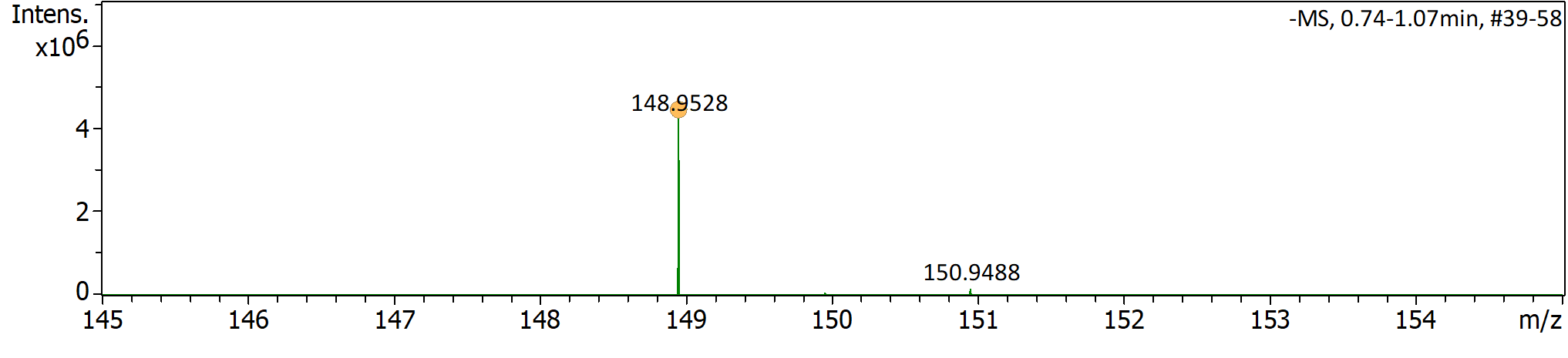

**UV-visible Absorption**

UV-visible absorption spectra of PCs, Iod, EDB and Sulfs dissolved in DCM (2×10^-5^ M), were recorded with a JASCO V730 spectrometer (1 cm optical path length), respectively. The molar extinction coefficient of each compound was calculated using the Lambert Beer's Law (see Equation (S1)).

$A=\varepsilon\times C\times L$ Equation S1

In Equation S1, A and C correspond to the absorbance and the concentration of each compound in DCM respectively. ε is the molar extinction coefficient and L is the optical path length controlled at 1 cm.

Steady-state photolysis of PC dissolved in DCM (2×10^-5^ M) was also performed on a JASCO V730 spectrometer, upon LED@405nm (110 mW.cm^-2^) irradiation. After that, steady-state photolysis experiments of PCs were performed on PC/Iod, PC/EDB, PC/Sulf, PC/Iod/EDB and PC/Sulf/EDB systems, respectively (PC 2×10^-5^ M, Iod 4×10^-5^ M, EDB 4×10^-5^ M, Sulf 4×10^-5^ M).

**Fluorescence Spectra and Lifetime**

Fluorescence emission spectra of PCs (2×10^-5^ M in DCM) were investigated on a JASCO FP-750 spectrofluorometer. Singlet excited state energy (Es1) of each compound was calculated with the following Equation S2.

$E_{S1}=\frac{1240 nm}{WL}\times23.06 \frac{Kcal}{mol}$ Equation S2

In Equation S2, WL is the x coordinate of the intersection without unit of normalized fluorescence emission spectra and the normalized UV visible absorption spectra.

Fluorescence excited-state lifetimes were estimated with a HORIBA PPD-850 detector. The colloidal silica suspension LUDOX was utilized to evaluate the impulse response function (IRF) and the excitation wavelength was set at 367 nm, and its excitation wavelength was set at 367 nm and the pulse duration was shorter than 1.4 ns.

For fluorescence quenching, the Stern-Volmer quenching constant (K_sv_) between PC and Iod, PC and EDB, PC and Sulf, were calculated from classical Stern-Volmer Equation S3:

${I_{0}}/I=1+K_{Additive}^{SV}\left[ Additive \right]$ Equation S3

Where I_0_ and I stand for the fluorescence intensity of the PC in the absence and presence of Iod, EDB and Sulf, respectively. Here, Iod, EDB and Sulf were seen as different additives.

Electron-transfer quantum yields were calculated using Equation S4:

$\Phi_{eT}=\frac{K_{Additive}^{SV}\left[ Additive \right]}{1+K_{Additive}^{SV}\left[ Additive \right]}$ Equation S4

The quenching constants (K_q_) were calculated from Equation S5:

$K_{Additive}^{SV}=k_{q}\times t_{0}$  Equation S5

Where t_0_ stands the lifetime of the PC in DCM.

**Electron Spin Resonance (ESR)**

ESR experiments were conducted using a X-band spectrometer (Bruker EMX plus) at room temperature. N_2_-saturated solution of PBN in tert-butylbenzene was used as the free radical trapping agent. Radicals were generated at room temperature upon exposure to LED@405nm (110 mW.cm^-2^).

**Cyclic Voltammetry**

The oxidation (E_ox_) and reduction (E_red_) potentials were determined by cyclic voltammetry (E/V vs. Ag/AgCl). PCs were co-dissolved with tetrabutylammonium hexafluorophosphate (as the supporting electrolyte) in DCM, respectively. In the literature, the reduction potential of Iod is -0.7 V vs. SCE^[10]^, while the oxidation potential of EDB is 1.1 V vs. SCE^[11]^. In order to facilitate a comparison, E_red_ of Iod and E_ox_ of EDB were adjusted to values relative to Ag/AgCl using Equation S6, i.e., E_red_ of Iod is -0.747 V vs. Ag/AgCl, and E_ox_ of EDB is 0.953 V vs. Ag/AgCl.

$E_{vsAg/AgCl}=E_{vs SCE}-0.047$ Equation S6

Free Radical Photopolymerization

The different photoinitiating systems and resin were stirred in glass bottles overnight away from light exposure. For PC/Iod/EDB topic, the concentration of PC, Iod, EDB in 1 g PEGDA was controlled at 1.0×10^-6^ mol, 2.0×10^-6^ mol and 2.0×10^-6^ mol, respectively. For PC/Sulf/EDB systems, the concentration of PC, Sulf, EDB in 1 g PEGDA was controlled at 1.0×10^-6^ mol, 2.0×10^-6^ mol and 2.0×10^-6^ mol, as well. Drops of homogenous formulations were deposited in a mold to prepare 1.4 mm thick sample. Then, the different formulations were exposed to the irradiation of a LED@405nm (110 mW.cm^-2^), a LED@450nm (70 mW.cm^-2^), a LED@470nm (45 mW.cm^-2^) and a LED@530nm (45 mW.cm^-2^) at room temperature. The characteristic peak of PEGDA for thick samples was selected at 6160 cm^-1^. During photopolymerization process, samples were placed in RT-FTIR device and then LEDs were switched after 10 s measuring time of the RT-FTIR device. The illumination time of the whole photopolymerization process was 790 s and the whole testing time was 800 s. The acrylate function conversion (FC) of resin was calculated by Equation S7:

${FC}_{(t)}=\frac{(A_{0}-A_{t})}{A_{0}}\times100\%$ Equation S7

In Equation S7, A_0_ and A_t_ are the peak area at 0 s and at any t s, respectively.

According to RTFTIR, the photopolymerization kinetic slop is equal to R_p_/[M_0_]x100, where [M_0_] is the initial monomer concentration. Therefore, the photopolymerization rate (R_p_) can be translated clearly by the slop of the RT-FTIR curves for photopolymerization.^[12, 13]^

3D Printing and Direct Laser Write (DLW)

For this experiment, only the formulations exhibiting a good photopolymerization performance were investigated.

PEGDA contraining BTXIO/Iod/EDB was the best candidate for 3D printing, which was performed on a 3D printer (Anycubic Photon D2) based on digital light processing (DLP). The printing parameters were set as follow: a layer thickness of 0.020 mm, a normal exposure time of 50 s, a bottom exposure time of 100 s, and five bottom layers.

For DLW process, the best condidate of PEGDA containing BTXI-Br/DBT/EDB, was deposited in a homemade glass tank (thickness 2 mm). Then, a laser diode@405nm controlled computer program was used as the light source for a spatial irradiation to manufacture specific 3D patterns (thickness 1.0 mm). The laser diode spot size and intensity were around 50 μm and 110 mW.

After printing, 3D structures were cleaned with ethanol or acetone to remove the uncured formulation part. After that, a scanning electron microscope (SEM) was used to observe the morphology of the surface of the 3D structures.


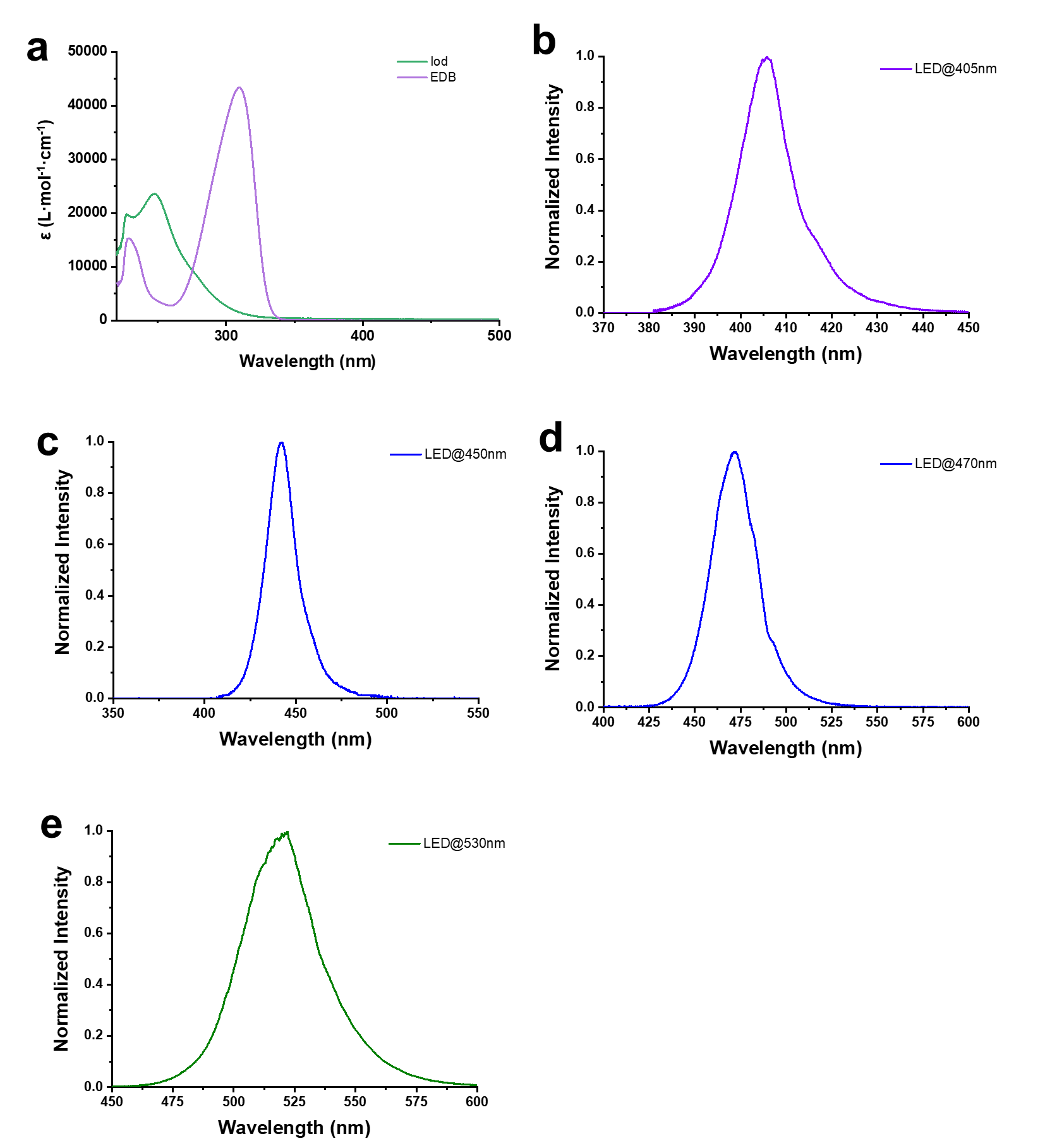


**Figure S1.** (a) UV-Visible spectra of Iod and EDB in DCM. The emission spectra of (b) LED@405nm, (c) LED@450nm, (d) LED@470nm, and (e) LED@530nm.


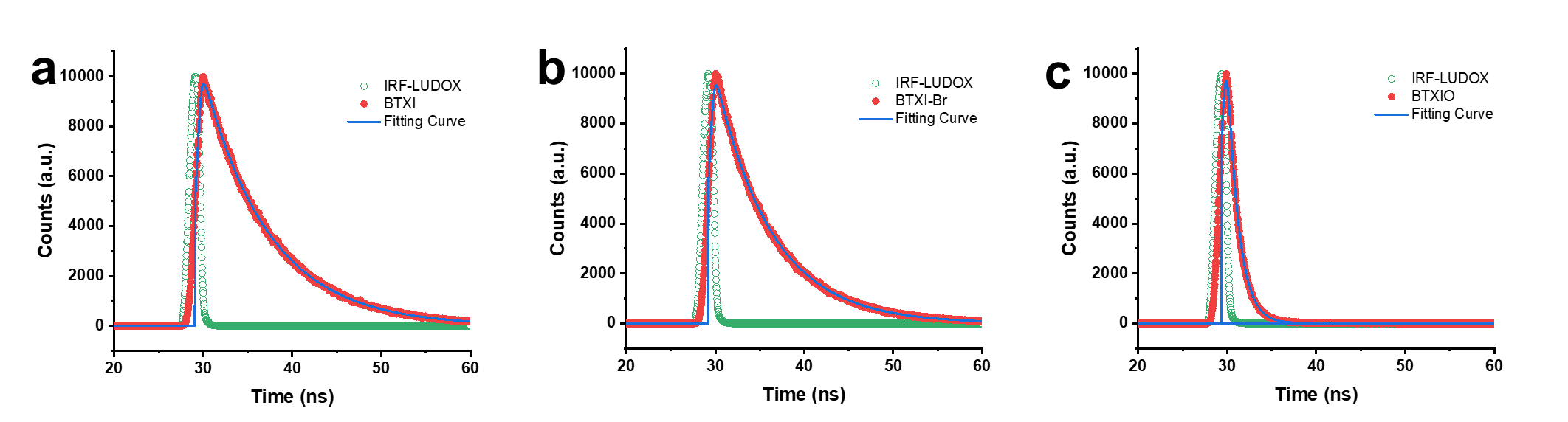


**Figure S2.** Lifetime of three PCs: (a) BTXI, (b) BTXI-Br and (c) BTXIO.


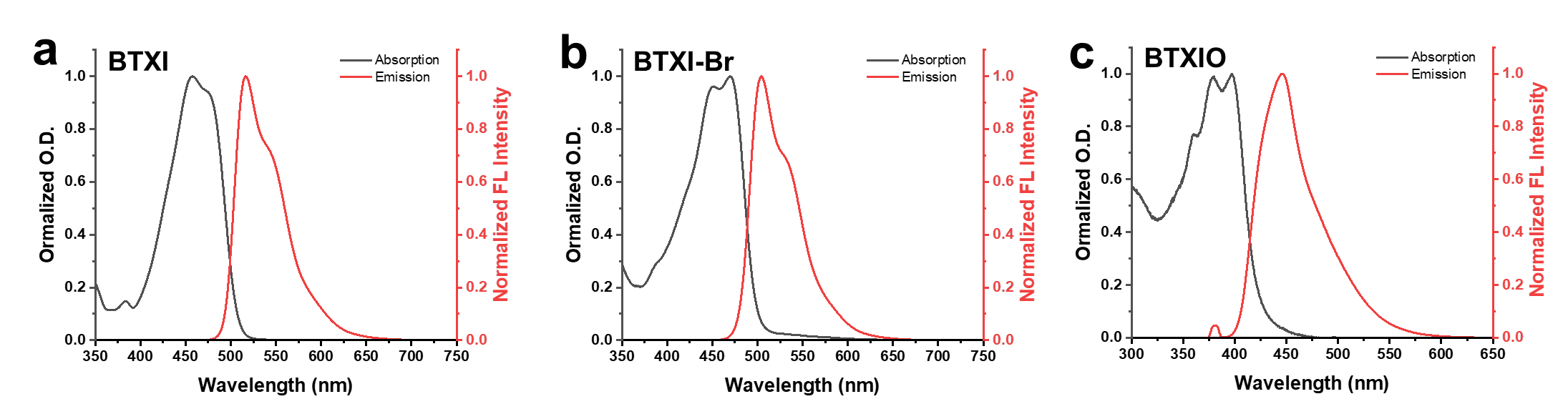


**Figure S3.** Singlet-state energy determination of (a) BTXI, (b) BTXI-Br and (c) BTXIO.


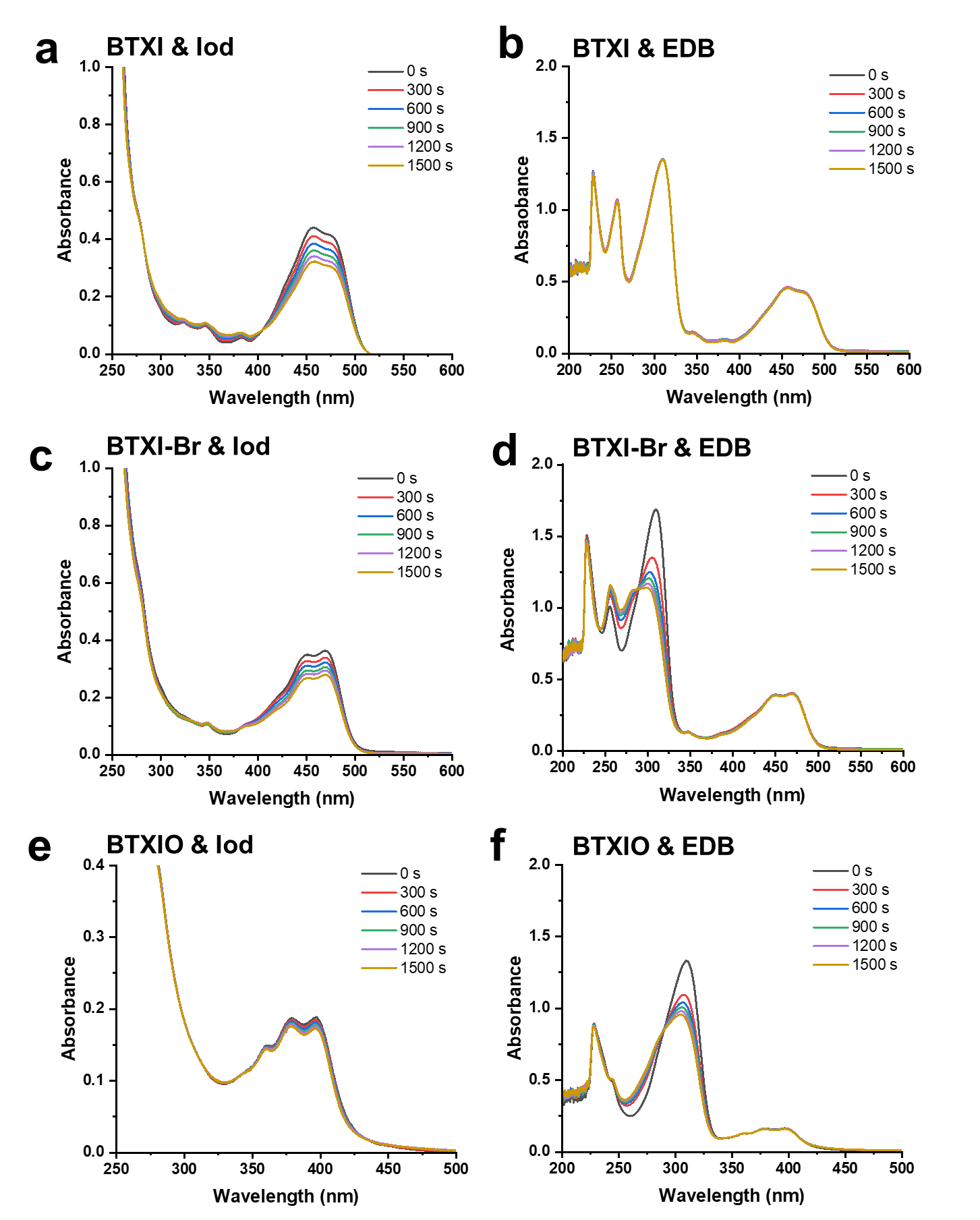


Figure S4. Photolysis of different photoinitiating systems in DCM under LED@405nm irradiation. (a) BTXI/Iod, (b) BTXI/EDB, (c) BTXI-Br/Iod, (d) BTXI-Br/EDB, (e) BTXIO/Iod, and (f) BTXIO/EDB. The concentration of PC, Iod and EDB in DCM was 2×10^-5^ M 4×10^-5^ M, EDB 4×10^-5^ M, respectively.

**
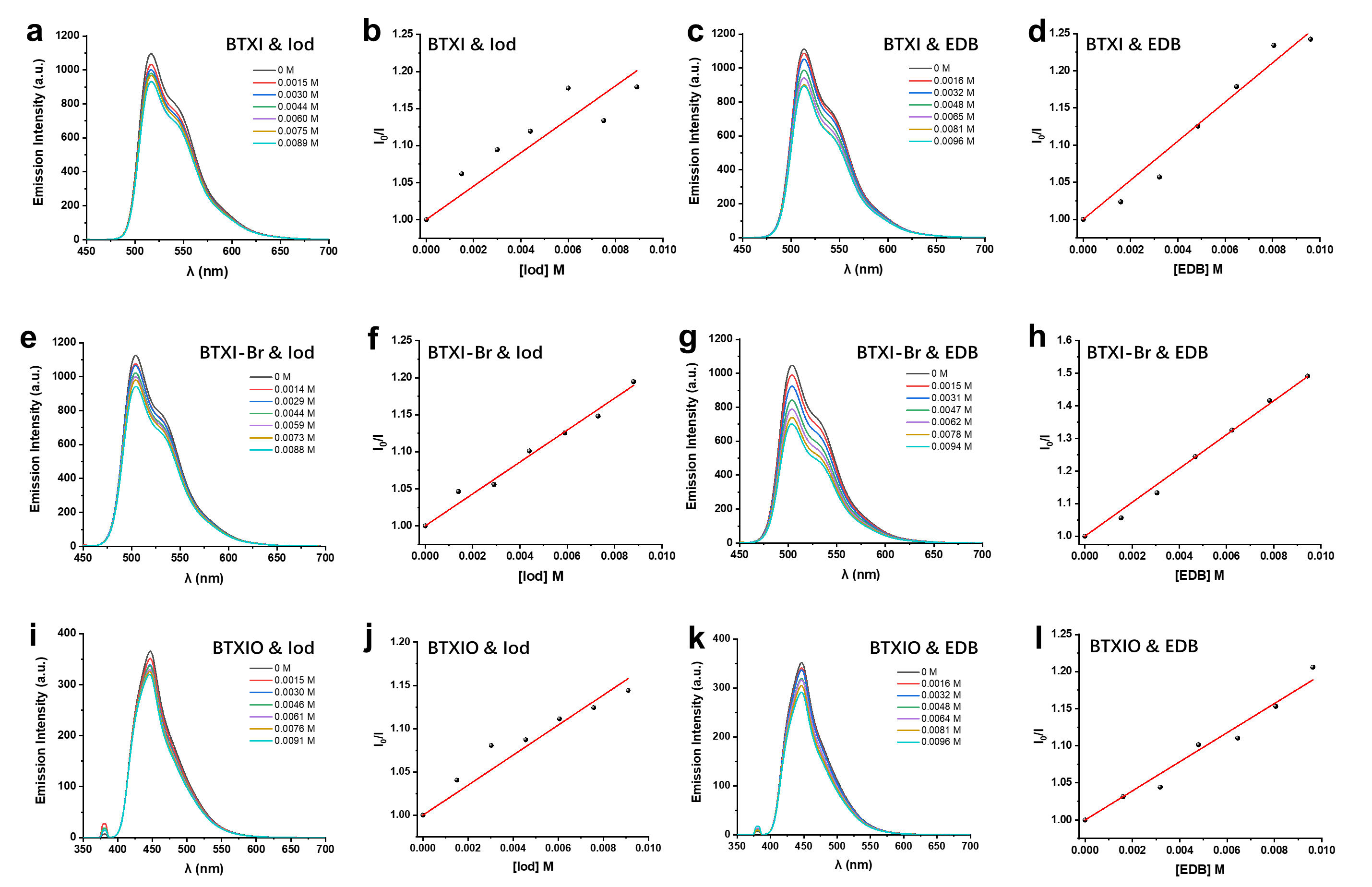
**

**Figure S5.** Fluorescence quenching of (a) BTXI/Iod, (c) BTXI/EDB, (e) BTXI-Br/Iod, (g) BTXI-Br/EDB, (i) BTXIO/Iod and (k) BTXIO/EDB. Stern-Volmer treatment for fluorescence quenching of (b) BTXI/Iod, (d) BTXI/EDB, (f) BTXI-Br/Iod, (h) BTXI-Br/EDB, (j) BTXIO/Iod and (l) BTXIO/EDB. The concentration of PC was 2×10^-5^ M in DCM.

**
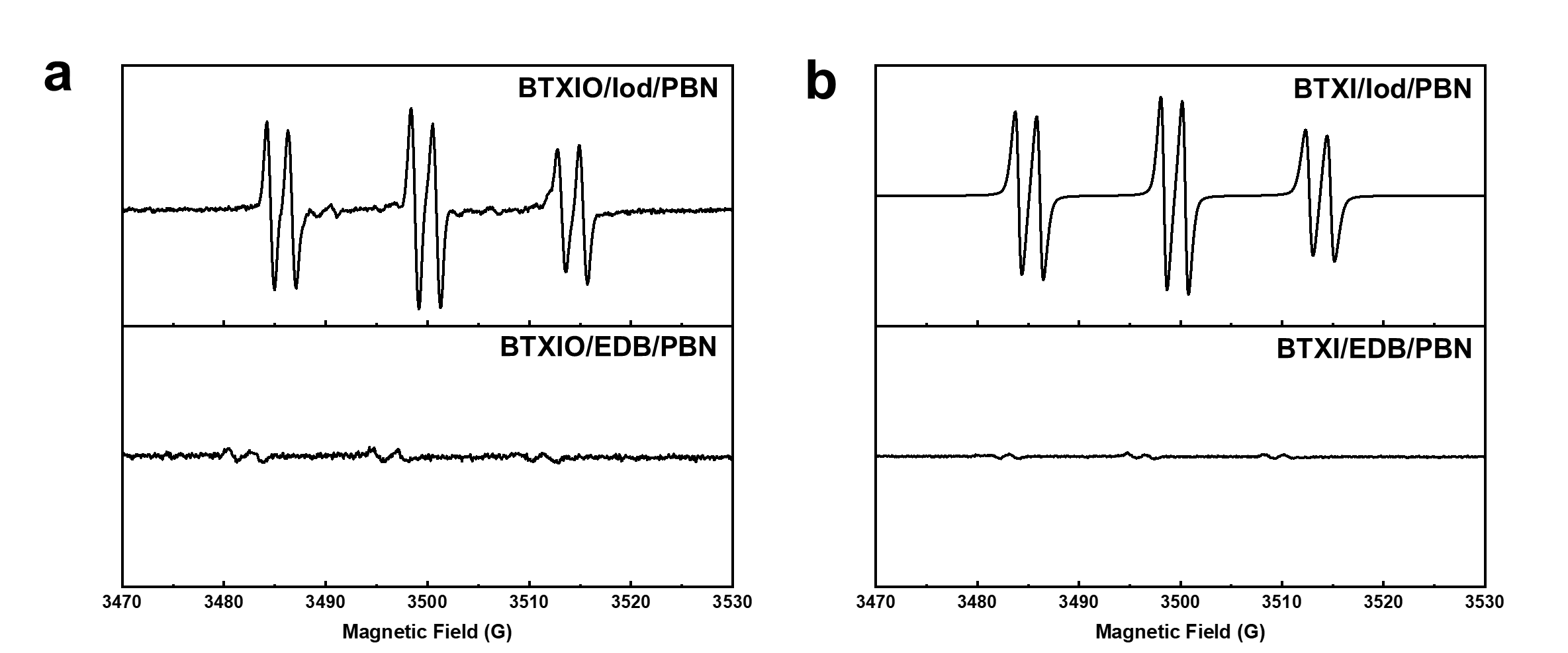
**

**Figure S6.** ESR of (a) BTXIO/Iod/PBN and BTXIO/EDB/PBN, and (b) BTXI/Iod/PBN and BTXI/EDB/PBN under the irradiation of LED@405nm after 100 s.

**
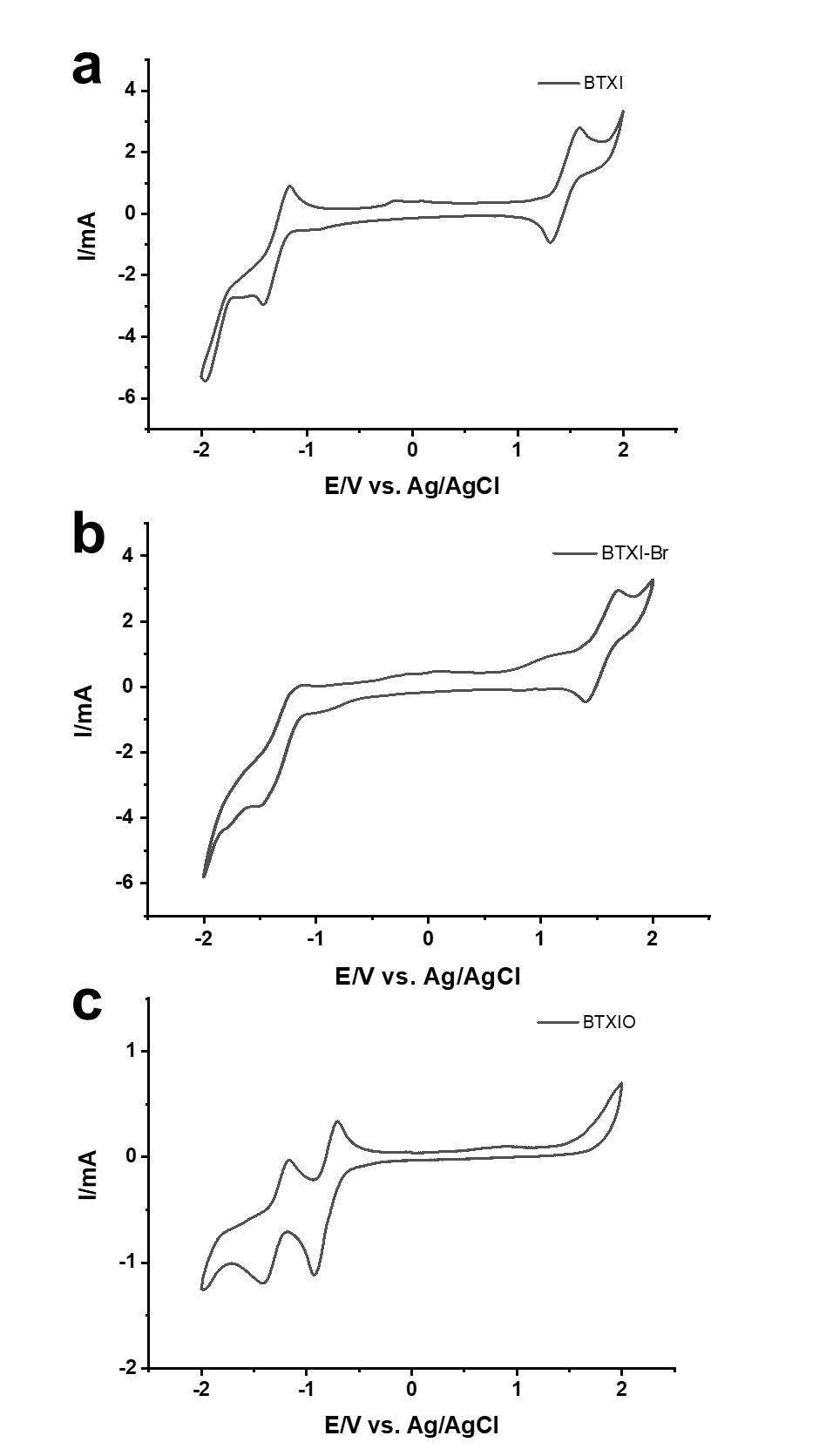
**

**Figure S7.** Cyclic voltammetry of (a) BTXI, (b) BTXI-Br and (c) BTXIO.

**
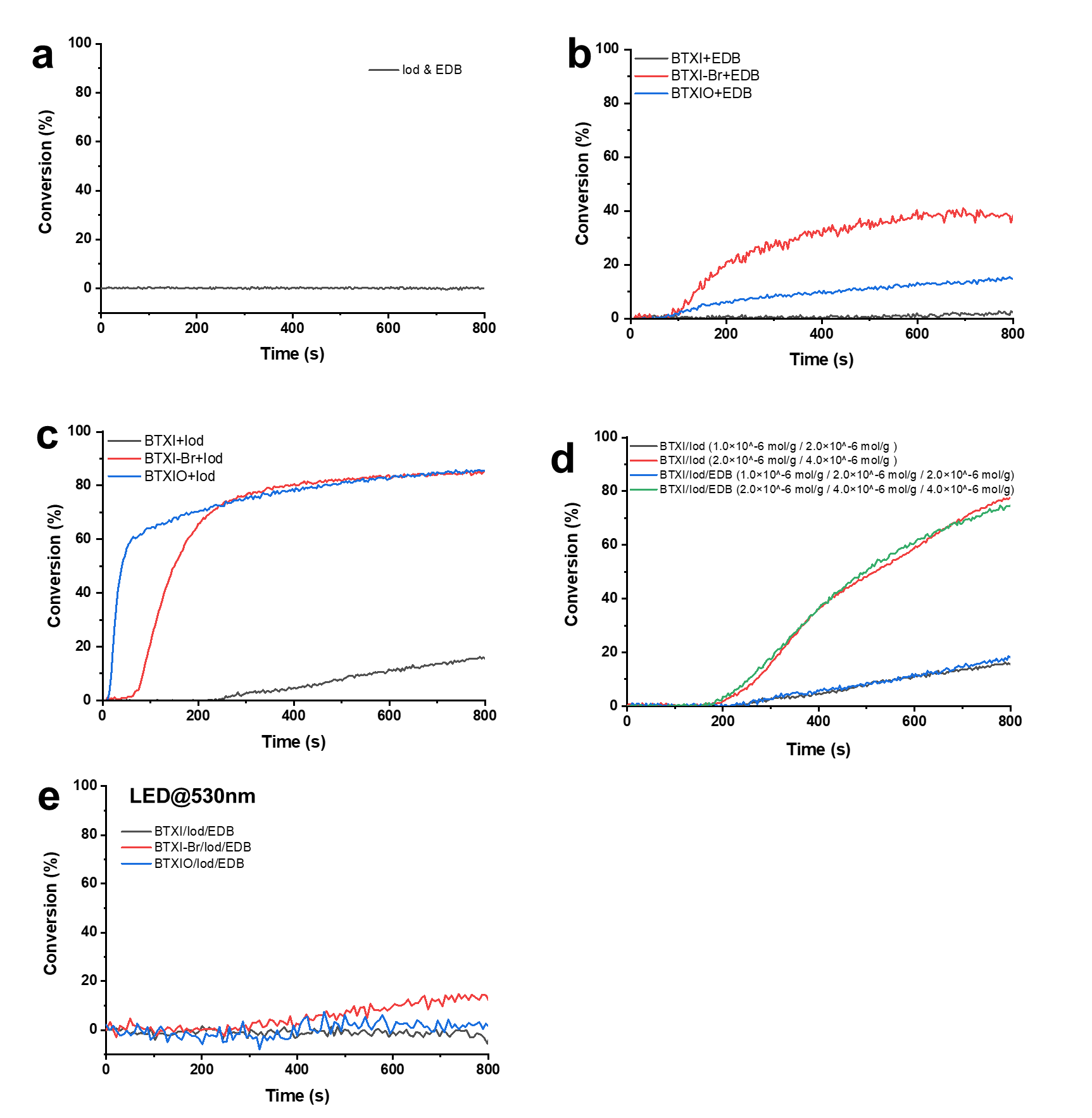
**

**Figure S8.** Photopolymerization of PEGDA initiated by (a) Iod/EDB at 405nm (EDB 2.0×10^-6^ mol and Iod 2.0×10^-6^ mol in 1 g PEGDA), (b) PC/EDB at 405 nm and (c) PC/Iod at 405 nm (The concentration of these photoinitiating systems, were controlled at PC, 1.0×10^-6^ mol, EDB 2.0×10^-6^ mol and Iod 2.0×10^-6^ mol in 1 g PEGDA, respectively.). (d) Photopolymerization of PEGDA initiated by BTXI/Iod and BTXI/Iod/EDB with different concentrations, (e) Photopolymerization of PEGDA initiated by PC/Iod/EDB under LED@530nm. Thickness of PEGDA sample was 1.4 mm.

**Table S1.** Acrylate function conversion (FC) and photopolymerization rate (R_p_) of PEGDA initiated by different photoinitiating systems under irradiation of LED@405nm.

| **PC** | **PC/Iod** | | **PC/EDB** | | **PC/Iod/EDB** | |
| --- | --- | --- | --- | --- | --- | --- |
|  | **FC (%, t= 800 s)** | **R_p_/[M_0_]x100 (s^-1^)** | **FC (%, t= 800 s)** | **R_p_/[M_0_]x100 (s^-1^)** | **FC (%, t= 800 s)** | **R_p_/[M_0_]x100 (s^-1^)** |
| **BTXI** | 16 | 0.1 | 3 | 0.1 | 19 | 0.1 |
| **BTXI-Br** | 85 | 0.8 | 41 | 0.5 | 91 | 1.3 |
| **BTXIO** | 86 | 2.6 | 15 | 0.2 | 91 | 3.7 |

**Table S2.** Acrylate function conversion (FC) and photopolymerization rate (R_p_) of PEGDA initiated by PC/Iod/EDB under irradiation of LED@450nm, LED@470nm and LED@530nm, respectively.

| **Light Source** | **PC/Iod/EDB** | **FC (%, t= 800 s)** | **R_p_/[M_0_]x100 (s^-1^)** |
| --- | --- | --- | --- |
| **LED@450nm** | **BTXI** | 0 | / |
|  | **BTXI-Br** | 89 | 0.6 |
|  | **BTXIO** | 91 | 1.9 |
| **LED@470nm** | **BTXI** | 0 | / |
|  | **BTXI-Br** | 72 | 0.2 |
|  | **BTXIO** | 69 | 0.3 |
| **LED@530nm** | **BTXI** | 0 | / |
|  | **BTXI-Br** | 11 | / |
|  | **BTXIO** | 0 | / |


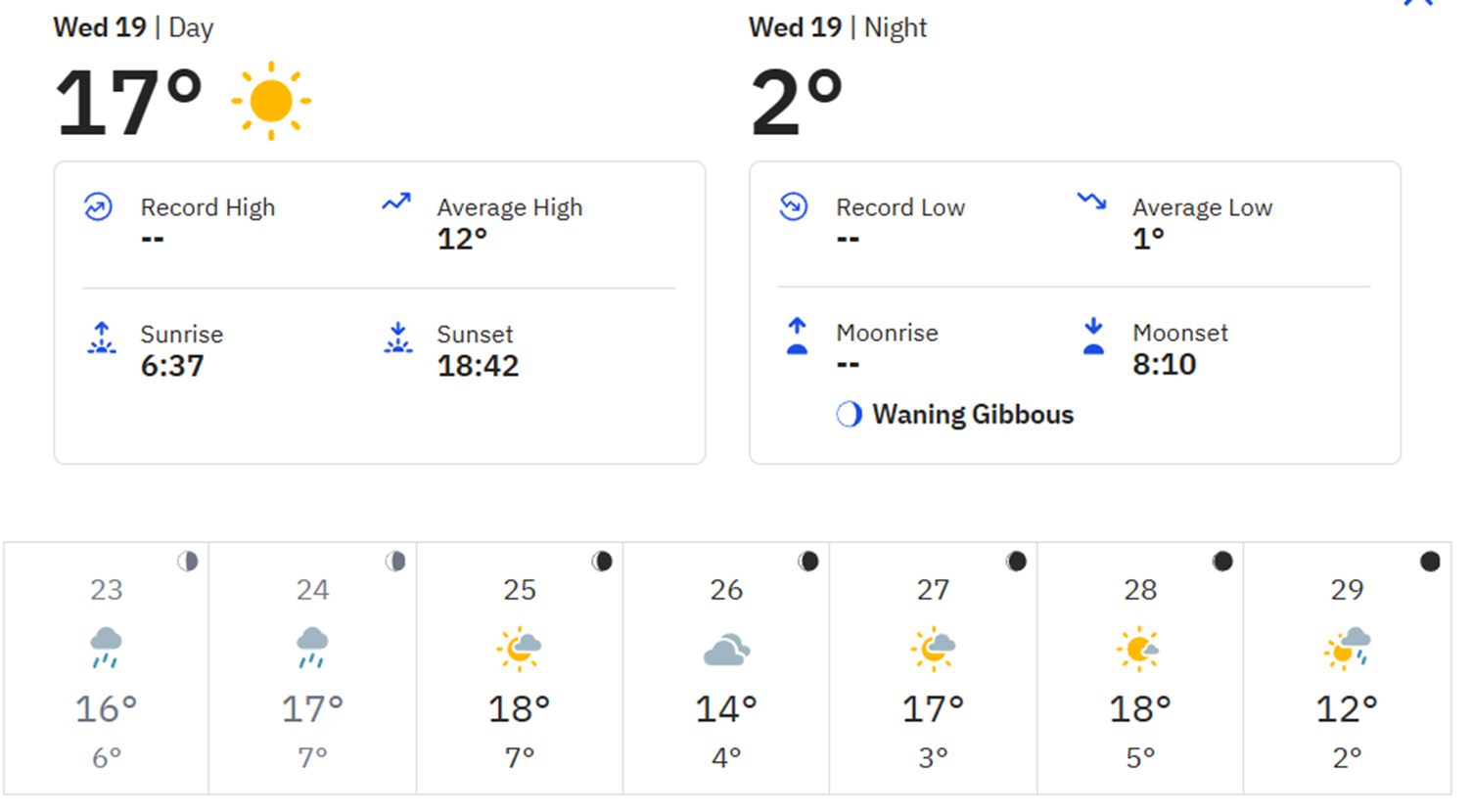


**Figure S9.** Weather report of March 19th 2025 in Mulhouse, France.


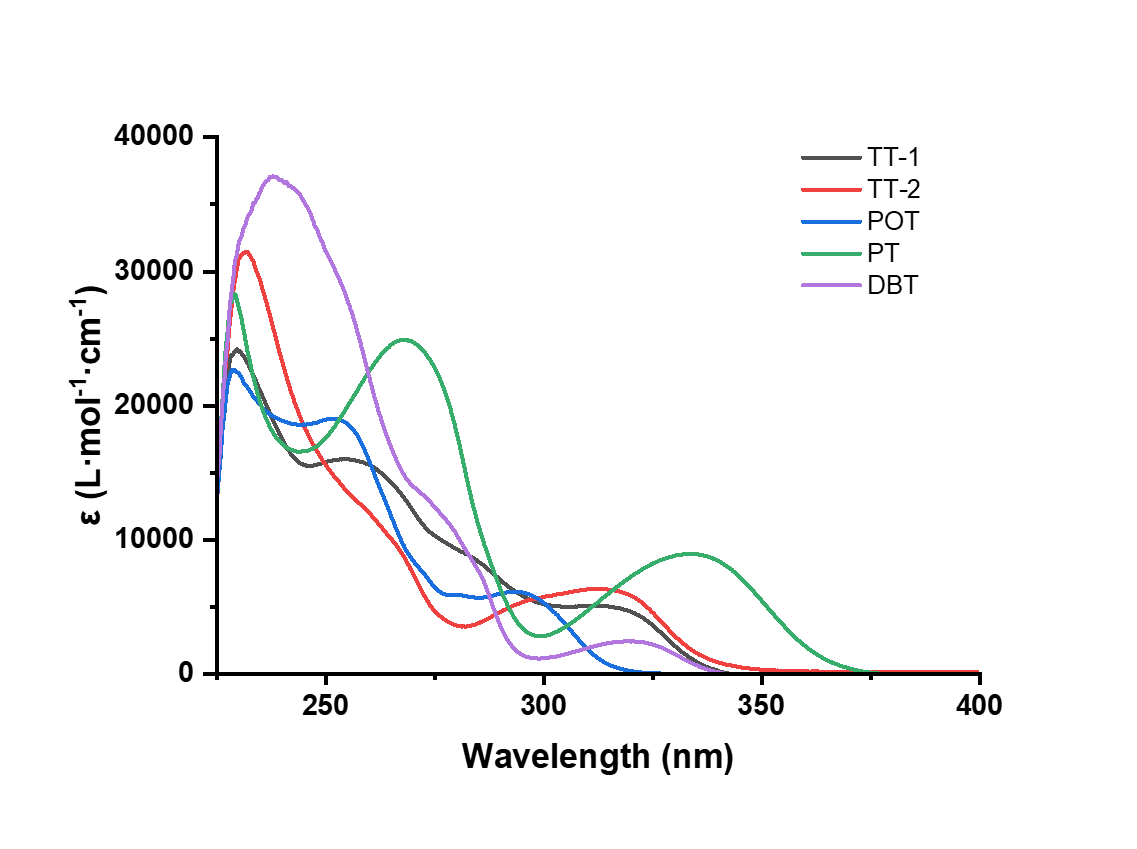


**Figure S10**. UV-visible spectra of five Sulfs.


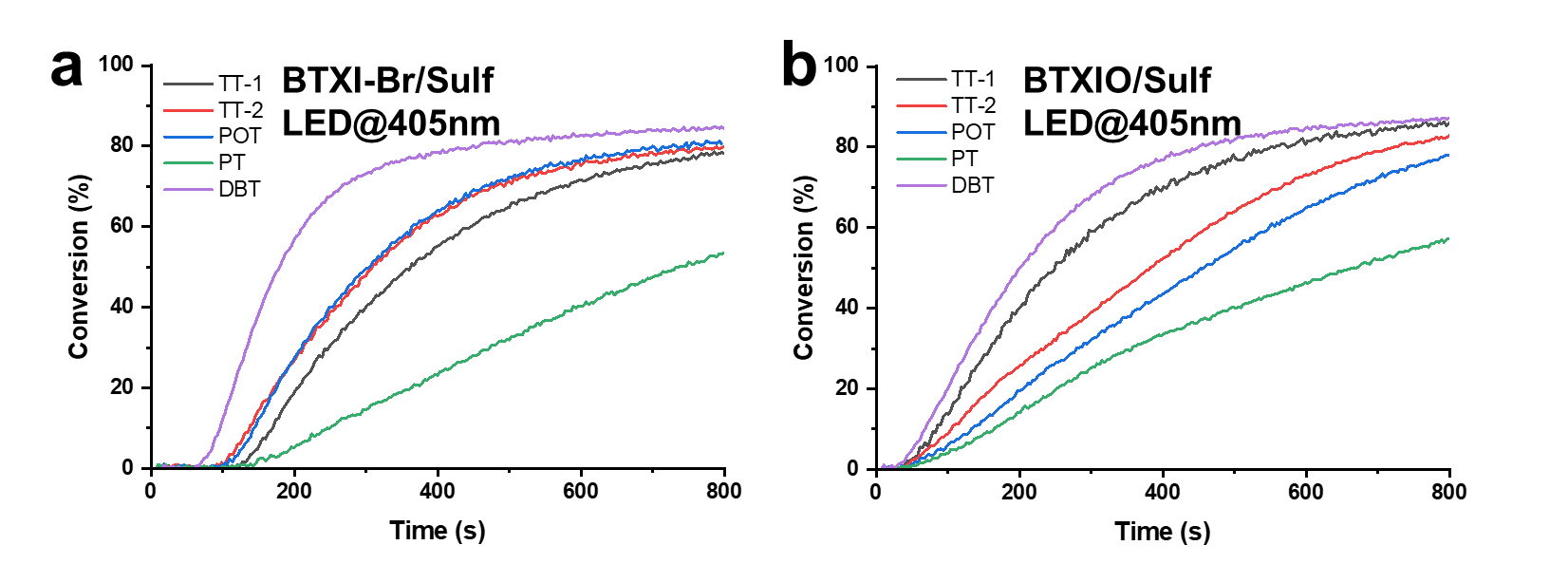


**Figure S11.** Photopolymerization of PEGDA initiated by (a) BTXI-Br/Sulf under LED@405nm, (b) BTXIO/Sulf under LED@405nm.

**Table S3.** Acrylate function conversion (FC) and photopolymerization rate (R_p_) of PEGDA initiated by different photoinitiating systems under irradiation of LED@405nm.

|  | **BTXI-Br/Sulf** | | **BTXI-Br/Sulf/EDB** | | **BTXIO/Sulf** | | **BTXIO/Sulf/EDB** | |
| --- | --- | --- | --- | --- | --- | --- | --- | --- |
|  | **FC**  **(%, t= 800 s)** | **R_p_/[M_0_]x100**  **(s^-1^)** | **FC**  **(%, t= 800 s)** | **R_p_/[M_0_]x100**  **(s^-1^)** | **FC**  **(%, t= 800 s)** | **R_p_/[M_0_]x100**  **(s^-1^)** | **FC**  **(%, t= 800 s)** | **R_p_/[M_0_]x100**  **(s^-1^)** |
| **TT-1** | 79 | 0.4 | 88 | 0.9 | 86 | 0.5 | 90 | 0.6 |
| **TT-2** | 80 | 0.4 | 90 | 1.5 | 83 | 0.3 | 88 | 0.5 |
| **POT** | 81 | 0.4 | 92 | 1.5 | 78 | 0.2 | 88 | 0.4 |
| **PT** | 54 | 0.3 | 76 | 0.4 | 58 | 0.2 | 76 | 0.4 |
| **DBT** | 85 | 0.6 | 92 | 1.7 | 87 | 0.4 | 91 | 0.8 |

**Table S4.** Acrylate function conversion (FC) and photopolymerization rate (R_p_) of PEGDA initiated by different photoinitiating systems under irradiation of LED@450nm.

|  | **BTXI-Br/Sulf/EDB** | | **BTXIO/Sulf/EDB** | |
| --- | --- | --- | --- | --- |
|  | **FC**  **(%, t= 800 s)** | **R_p_/[M_0_]x100**  **(s^-1^)** | **FC**  **(%, t= 800 s)** | **R_p_/[M_0_]x100**  **(s^-1^)** |
| **TT-1** | 85 | 0.6 | 76 | 0.5 |
| **TT-2** | 84 | 0.6 | 63 | 0.3 |
| **POT** | 86 | 0.6 | 55 | 0.3 |
| **PT** | 26 | 0.3 | 39 | 0.3 |
| **DBT** | 89 | 0.6 | 79 | 0.5 |


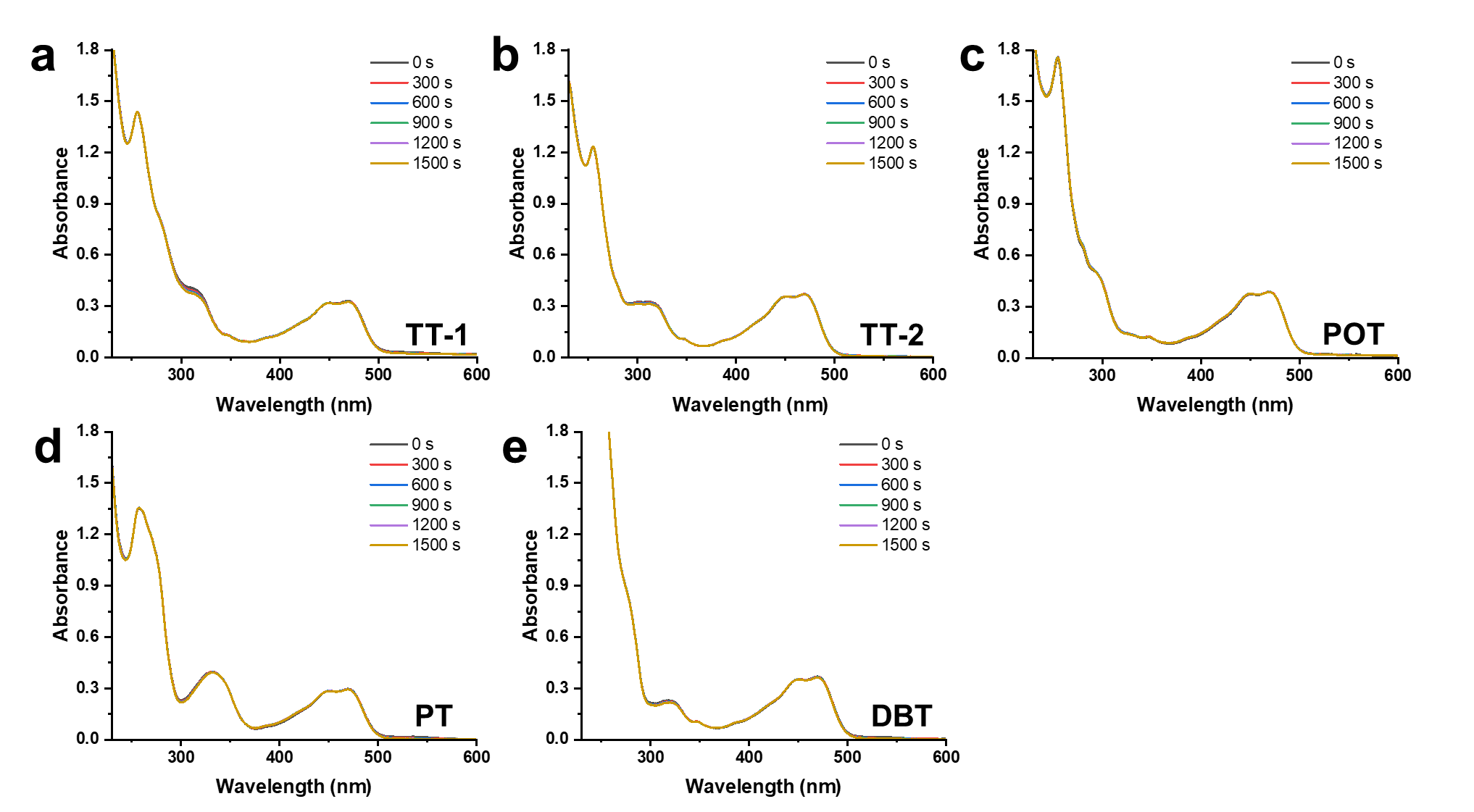


Figure S12. Photolysis of different two-component systems in DCM under LED@405nm irradiation. (a) BTXI-Br/TT-1, (b) BTXI-Br/TT-2, (c) BTXI-Br/POT, (d) BTXI-Br/PT, (e) BTXI-Br/DBT.


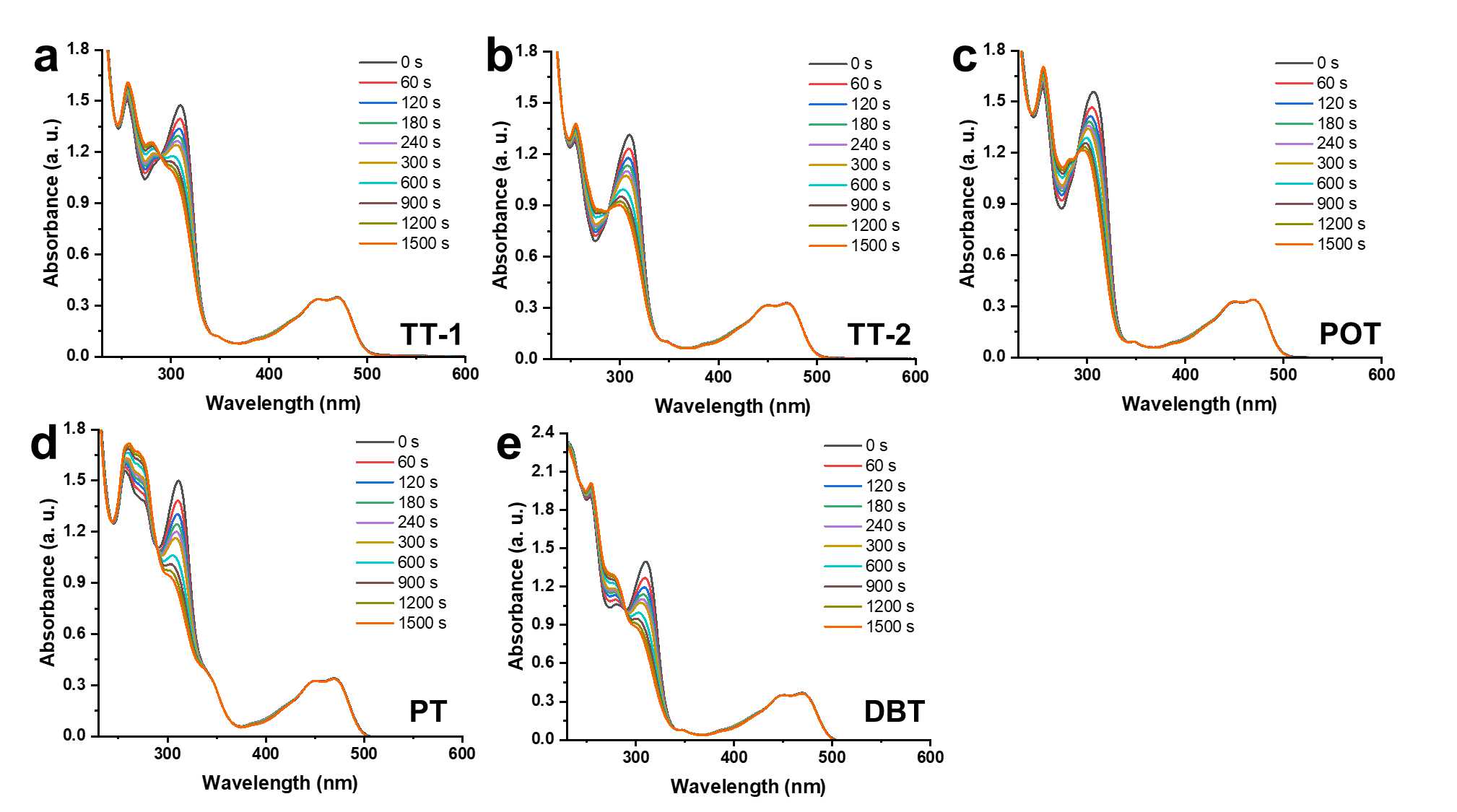


**Figure S13.** Photolysis of different three-component systems in DCM under LED@405nm irradiation. (a) BTXI-Br/TT-1/EDB, (b) BTXI-Br/TT-2/EDB, (a) BTXI-Br/TT-3/EDB, (d) BTXI-Br/POT/EDB, (e) BTXI-Br/PT/EDB, (f) BTXI-Br/DBT/EDB.


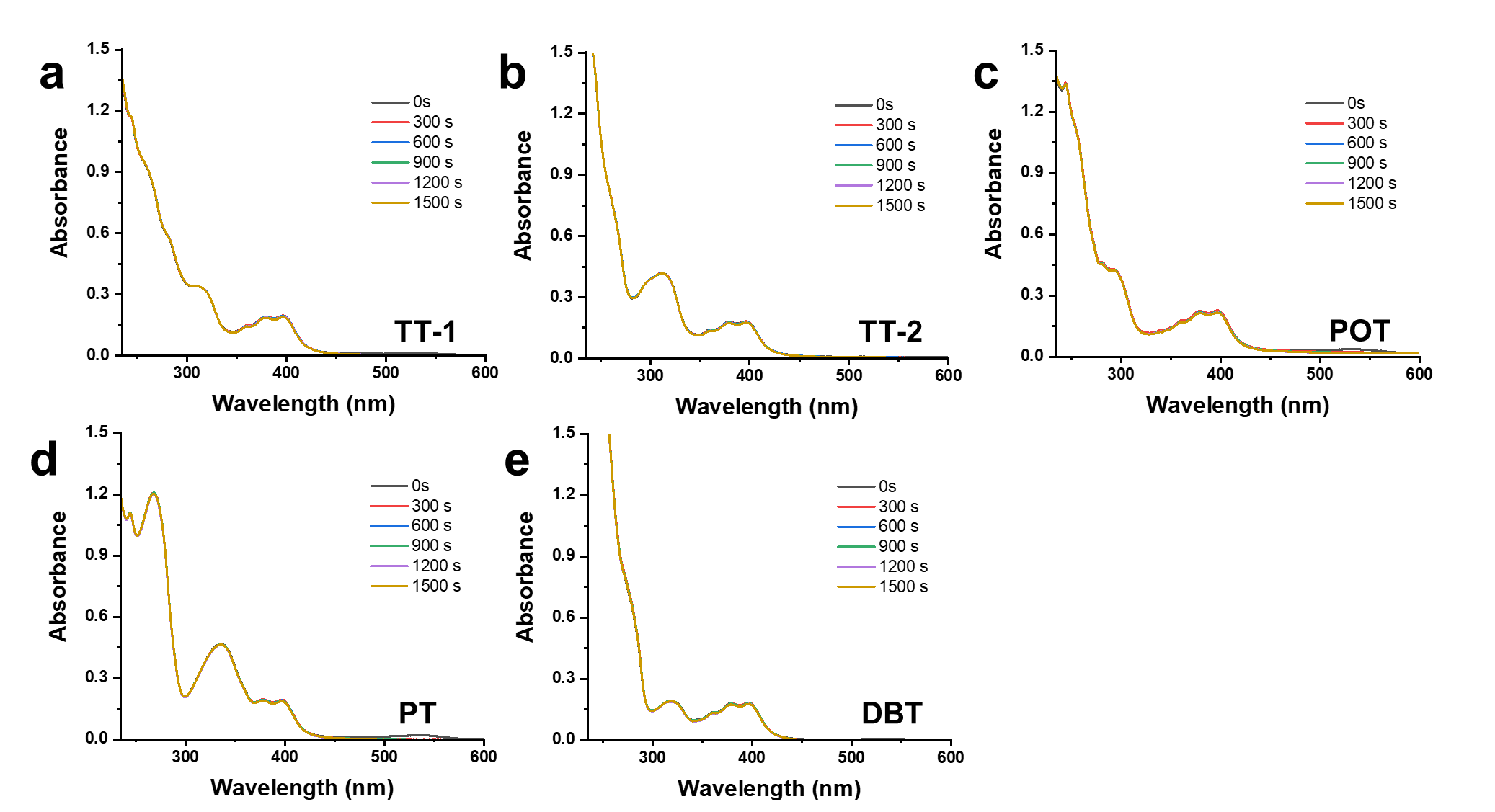


**Figure S14.** Photolysis of different two-component systems in DCM under LED@405nm irradiation. (a) BTXIO/TT-1, (b) BTXIO/TT-2, (c) BTXIO/POT, (d) BTXIO/PT, (e) BTXIO/DBT.


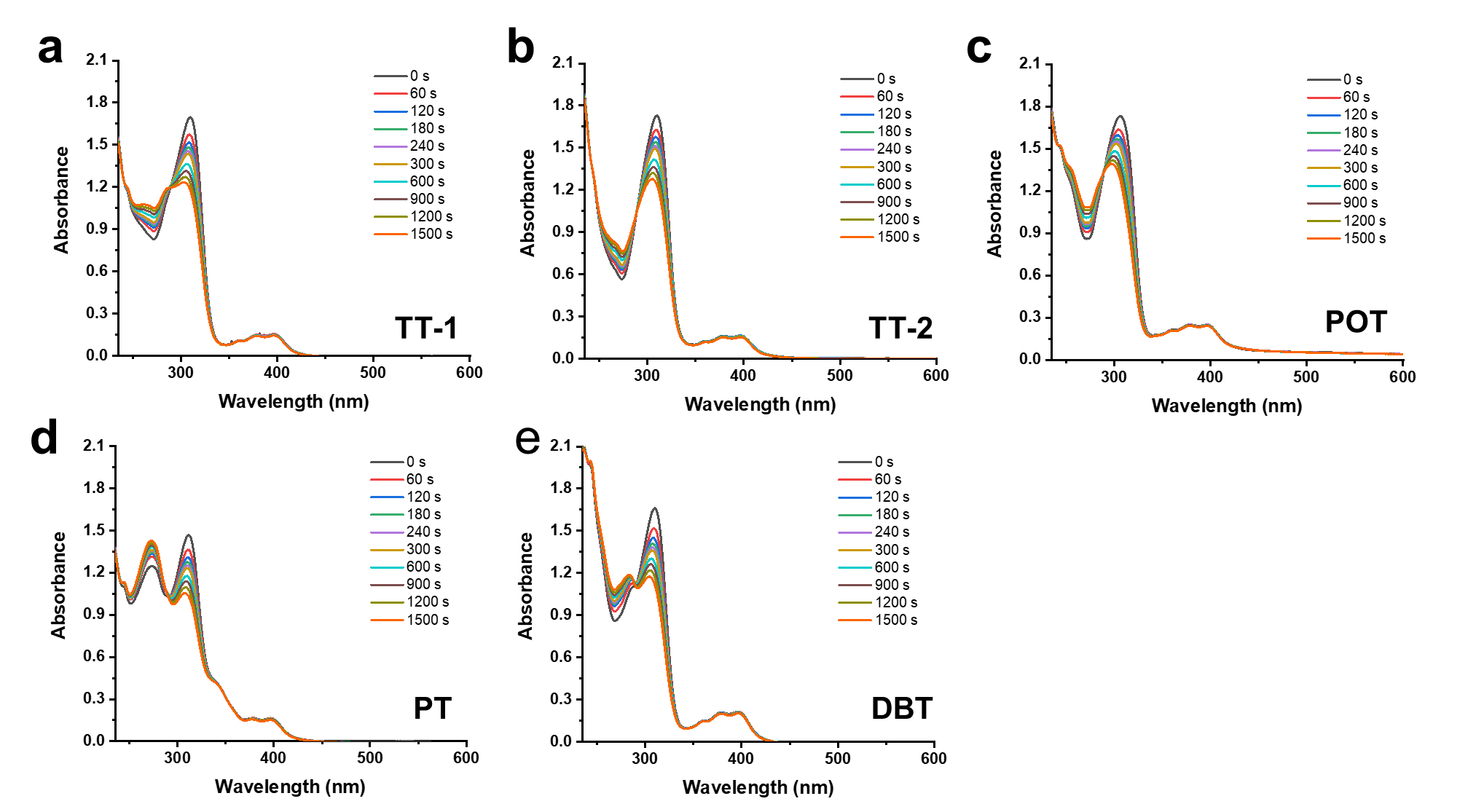


**Figure S15.** Photolysis of different three-component systems in DCM under LED@405nm irradiation. (a) BTXIO/TT-1/EDB, (b) BTXIO/TT-2/EDB, (c) BTXIO/POT/EDB, (d) BTXIO/PT/EDB, (e) BTXIO/DBT/EDB.


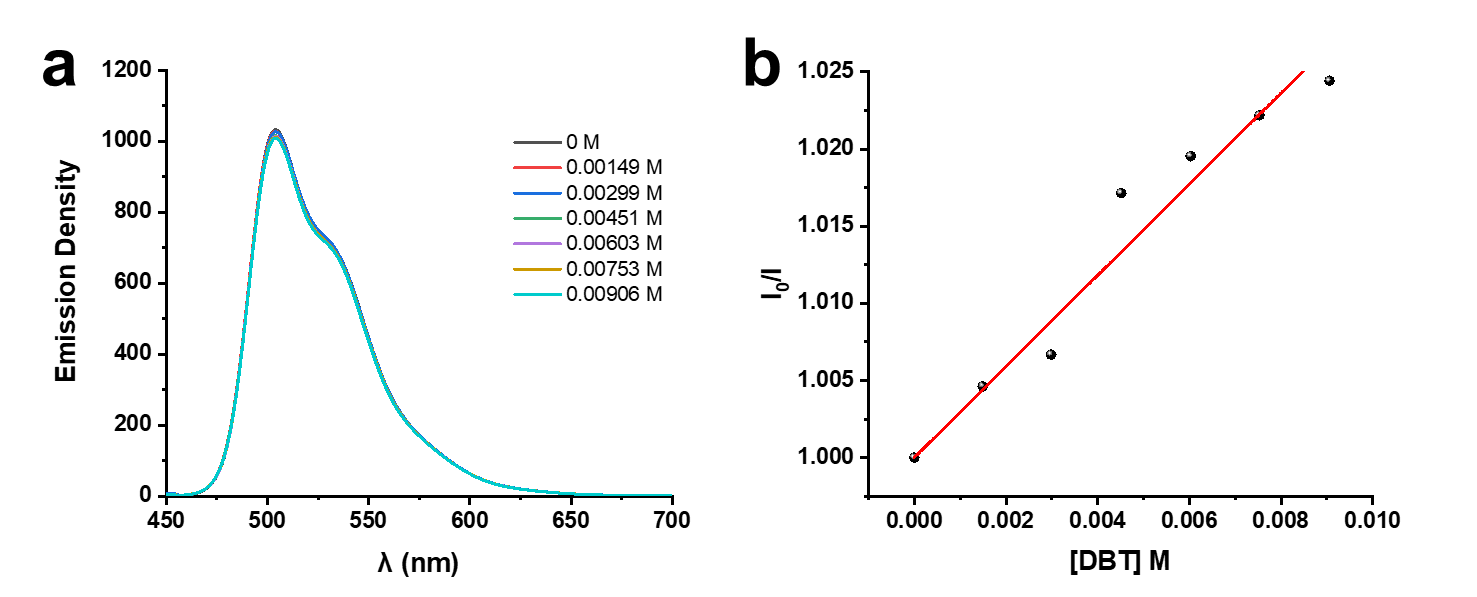


**Figure S16.** Fluorescence quenching of (a) BTXI-Br/DBT and Stern-Volmer treatment for fluorescence quenching of (b) BTXI/DBT.

**Table S5.** K^SV^_DBT_ of BTXI-Br.

| **PC** | **K^SV^_DBT_ [M^-1^]** |
| --- | --- |
| **BTXI-Br** | **3** |

**Reference**

[1] Y. Zhang, B. Song, Z. Liu, A. Noon, C. Dietlin, F. Morlet-Savary, M. Schmitt, D. Gigmes, F. Dumur, J. Lalevee, Angew. Chem. Int. Ed. Engl. **2024**, 63, e202405337-e202405346.

[2] T.-L. Ho, Synthesis **1973**, 1973, 347-354.

[3] K. Liu, J. Meng, X. Jiang, Org. Process Res. Dev. **2023**, 27, 1198-1202.

[4] P. Dao, F. Ye, Y. Liu, Z. Y. Du, K. Zhang, C. Z. Dong, B. Meunier, H. Chen, ACS Chem. Neurosci. **2017**, 8, 798-806.

[5] T. Yoshida, Y. Honda, T. Morofuji, N. Kano, Org. Lett. **2021**, 23, 9664-9668.

[6] J. Kirschner, F. Szillat, M. Bouzrati-Zereteli, J. M. Becht, J. E. Klee, J. Lalevée, Dent. Mater. **2020**, 36, 187-196.

[7] B. Zhao, Q. Wang, T. Zhu, B. Feng, M. Ma, Org. Lett. **2022**, 24, 5608-5613.

[8] A. Dewanji, L. van Dalsen, J. A. Rossi-Ashton, E. Gasson, G. E. M. Crisenza, D. J. Procter, Nat. Chem. **2023**, 15, 43-52.

[9] L. Shan, Z. Ma, C. Ou, Y. Cai, Y. Ma, Y. Guo, X. Ma, C. Liu, Org. Biomol. Chem. **2023**, 21, 3789-3793.

[10] P. P. Romańczyk, S. S. Kurek, Electrochim. Acta **2017**, 255, 482-485.

[11] F. Jean-Pierre, J. Lalevée, *Photoinitiators for polymer synthesis: scope, reactivity, and efficiency*, John Wiley & Sons, 2012.

[12] M. Bouzrati-Zerelli, J. Kirschner, C. P. Fik, M. Maier, C. Dietlin, F. Morlet-Savary, J. P. Fouassier, J.-M. Becht, J. E. Klee, J. Lalevée, Macromolecules **2017**, 50, 6911-6923.

[13] S. Liu, N. Giacoletto, M. Schmitt, M. Nechab, B. Graff, F. Morlet-Savary, P. Xiao, F. Dumur, J. Lalevée, Macromolecules **2022**, 55, 2475-2485.
